# Profiling microRNAs in lung tissue from pigs infected with *Actinobacillus pleuropneumoniae*

**DOI:** 10.1186/1471-2164-13-459

**Published:** 2012-09-06

**Authors:** Agnieszka Podolska, Christian Anthon, Mads Bak, Niels Tommerup, Kerstin Skovgaard, Peter MH Heegaard, Jan Gorodkin, Susanna Cirera, Merete Fredholm

**Affiliations:** 1 Department of Veterinary Clinical and Animal Sciences, Section of Anatomy, Cell Biology, Genetics and Bioinformatics, University of Copenhagen, Faculty of Health and Medical Sciences, Copenhagen, Denmark; 2 Biotech Research & Innovation Centre (BRIC), University of Copenhagen, Copenhagen, Denmark; 3 Center for non-coding RNA in Technology and Health, University of Copenhagen, Copenhagen, Denmark; 4 Department of Cellular and Molecular Medicine, Panum Institute, Copenhagen, Denmark; 5 Innate Immunology Group, National Veterinary Institute, Technical University of Denmark, Copenhagen, Denmark

**Keywords:** MicroRNA, RNAseq, High throughput sequencing, RT-qPCR, Actinobacillus pleuropneumoniae, Pig, Pleuropneumonia

## Abstract

**Background:**

MicroRNAs (miRNAs) are a class of non-protein-coding genes that play a crucial regulatory role in mammalian development and disease. Whereas a large number of miRNAs have been annotated at the structural level during the latest years, functional annotation is sparse. *Actinobacillus pleuropneumoniae* (APP) causes serious lung infections in pigs. Severe damage to the lungs, in many cases deadly, is caused by toxins released by the bacterium and to some degree by host mediated tissue damage. However, understanding of the role of microRNAs in the course of this infectious disease in porcine is still very limited.

**Results:**

In this study, the RNA extracted from visually unaffected and necrotic tissue from pigs infected with *Actinobacillus pleuropneumoniae* was subjected to small RNA deep sequencing. We identified 169 conserved and 11 candidate novel microRNAs in the pig. Of these, 17 were significantly up-regulated in the necrotic sample and 12 were down-regulated. The expression analysis of a number of candidates revealed microRNAs of potential importance in the innate immune response. MiR-155, a known key player in inflammation, was found expressed in both samples. Moreover, miR-664-5p, miR-451 and miR-15a appear as very promising candidates for microRNAs involved in response to pathogen infection.

**Conclusions:**

This is the first study revealing significant differences in composition and expression profiles of miRNAs in lungs infected with a bacterial pathogen. Our results extend annotation of microRNA in pig and provide insight into the role of a number of microRNAs in regulation of bacteria induced immune and inflammatory response in porcine lung.

## Background

MicroRNAs (miRNAs) are approximately 22 nucleotide (nt) long molecules, constituting a highly abundant class of non-coding RNAs (ncRNAs) encoded in the genome of all eukaryotes [1]. Over the last few years thousands of microRNAs have been discovered in different organisms and the conservation of most of the microRNAs has been confirmed across species [2], [3]. This novel class of ncRNAs provides a new, exciting view on gene regulation mechanism at the post transcriptional level by down-regulating their target mRNAs [4]. Finely tuned gene expression is crucial to physiological processes like embryonic development, cellular differentiation, cellular growth control, homeostasis and response to external stress factors such as pathogens. With a growing number of studies, it is becoming more apparent that microRNAs play a pivotal role in various physiological and developmental processes [5]. Some microRNAs are differentially expressed in a developmental-stage–specific, tissue-specific or pathological-stage-specific manner which is consistent with their gene regulatory function, while others seem to be present constitutively, suggesting their role in homeostasis mechanisms [6]. An increasing number of studies report the crucial role of microRNAs in human disorders and disease progression. De-regulation of many microRNA species has been associated with pathological states including cancer, neurodegenerative disorders, diabetes, bacterial infections and immunological response to the latter [5].

In protection against intruding pathogens, an organ-specific as well as a systemic immunological host response is activated. This response is activated by the presence of so called pathogen-associated molecular patterns (PAMPs) that are sensed by Pattern Recognition Receptors (PRRs), for instance the Toll-like receptor (TLR) [7]. Stimulation of PRRs leads to activation of the innate and adaptive immune response, elimination of the detected invading pathogen and development of a long lasting immunity against it. This complex and highly organized line of defense requires precisely balanced and well managed regulatory events for its proper function. Imbalanced inflammatory response may lead to septic shock-like condition that can cause death of the host due to multiple organ failure [8]. The expression of microRNAs involved in host response to pathogens is well established in viral infections [9], [10]. MicroRNAs like miR-29a and miR-32 have been found to repress the expression of viral mRNAs by possible recognition and targeting of viral nucleic acids with miR-29 specifically targeting HIV-1 3’UTR region [11], [12]. In contrast, replication of HCV (hepatitis C virus) is dependent on the activity of miR-122 [13]. Nevertheless, the role of mammalian microRNAs in bacterial infections is still in its infancy. There is evidence indicating strong involvement of microRNAs in immune response and inflammation after bacterial infection [14], [15]. TLRs recognizing PAMPs have been found to regulate several microRNAs. For example, miR-146a/b and miR-155 have been induced by TLR4-mediated sensing of bacterial lipopolysaccharide (LPS) [16], [17]. It has been shown that the nuclear factor kappa-light-chain-enhancer of activated B cells (NF-κB), a central transcription factor for a wide range of innate immune factors including several cytokines, interacts with the promoter region of miR-146a [16]. LPS stimulation results in up regulation of miR-155 expression. This microRNA is also believed to be under direct regulation of NF-κB and simultaneously regulating it [18]. Moreover, miR-155 has been shown to be induced by the bacterium *Helicobacter pylori*, a known pathogen of the human stomach [19] and has been proven to act as a global immune regulator in endotoxin-tolerant macrophages, proving that microRNA expression depends strongly on the status of infected cells [20].

*Actinobacillus pleuropneumoniae* (APP), serotype 5b is a bacterial pathogen infecting the porcine respiratory track, causing pleuropneumonia [21]. The disease causes severe economic losses to the pig industry. Very important virulence-associated factors of APP are the three different exotoxins belonging to the RTX family (Repeat in the structural ToXin, a family of exotoxins produced by gram-negative bacteria). The toxins cause serious damage to the lungs and interact with host immunity. The bacterium is represented by at least 12 different serotypes. Serotypes 1, 5, 9, 11, and 12 are usually highly virulent [22]. The complete picture of APP pathogenesis and host response to the infection has not yet been unraveled. There is a considerable lack of knowledge about the role that microRNAs play in APP infection. To our knowledge this is the first study on microRNA expression profiles in porcine lung tissue infected with *Actinobacillus pleuropneumoniae.* However, the expression profiles of protein coding genes in APP infection have been studied previously by [23].

The pig (*Sus scrofa*) has a high economic value for meat production worldwide and is an important animal model for biomedical research. The availability of a nearly completed assembly of the porcine genome allows annotation of various classes of ncRNAs and further provides possibilities for assigning function of those key molecules (microRNA) to the gene regulatory network. Yet, since the early studies of miRNAs in the pig genome [24,25], the number of porcine microRNAs deposited in miRBase [26-29] is considerably smaller than the number of miRNAs in human or mouse. The newest version of miRBase 18.0 includes 1727 microRNA entries in *Homo Sapiens*, 741 in *Mus musculus and only* 228 in *Sus scrofa.* More studies on porcine microRNAs are required for expansion of the repertoire of these small regulatory elements involved in development, growth and pathological conditions. The present study utilizes high throughput sequencing technology as well as bioinformatic tools to obtain expression profiles of microRNAs in porcine lung samples representing necrotic and visually unaffected areas, 14–18 h after experimental infection with *Actinobacillus pleuropneumoniae*. The above mentioned approaches to small RNA expression profiling, discovery and target predictions are reviewed in [30]. We detected a significant de-regulation of a number of host microRNAs during bacterial infection. A large number of those de-regulated microRNAs have putative target sites in genes involved in acute phase response; LPS induced innate immunity response to pathogens and apoptosis. The majority of the microRNA candidates selected for validation in the present study are found to target multiple (more than 10 different) protein coding genes. Moreover, several novel microRNAs have been discovered including one microRNA showing particularly high expression (i.e., included in top 20 most expressed microRNAs) in porcine lung. Interestingly, this putative novel microRNA appears to be specific for pig and closely related species as cow and dolphin. This is the first study elucidating the expression of microRNAs and their regulatory networks in pigs exposed to bacterial pathogen infection. Taken together, our data suggest that bacterial infection causes significant changes in the expression profiles of microRNAs within the infected lung, thus affecting the regulation of genes involved in the immunological response to inflammation and apoptosis.

## Results

### Overview of high throughput sequencing, alignment and clustering

**Figure 1:**
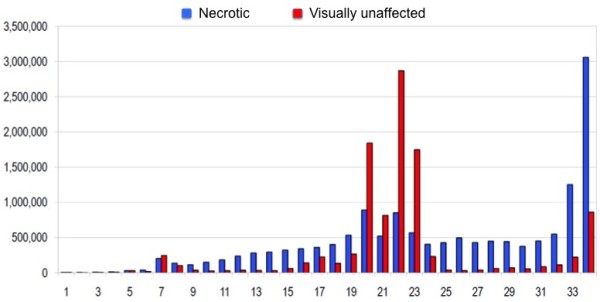
**The distribution of raw reads versus read lengths in both libraries.** X axis shows the insert length while Y axis represents raw reads. Necrotic area sample is marked in blue whereas visually unaffected area sample is marked in red.

To investigate the composition and dynamic changes of ncRNA (miRNA in particular) expression in lung tissue from pigs infected with APP*,* we constructed two small RNA libraries that were sequenced using Illumina GAIIx high throughput sequencing technology. A pool of eight samples from necrotic areas was used to create the necrotic library and the unaffected library was constructed out of ten pooled samples from visually unaffected areas. Illumina GAIIx sequencing generated 15,034,867 raw reads (un-normalized reads) in the necrotic sample and 12,544,524 raw reads in the visually unaffected sample, after filtering of low quality reads (Chastity > 0.6). For the necrotic sample, sub sequential adapter removal and quality filtering, resulted in a total of 11,997,185 18–34 nt long reads. Only high quality reads where accepted for alignment by Novoalign [31]. In the same sample, a total of 10,506,718 raw reads aligned to the pig genome [32] version 9 and 7,862,371 of these aligned uniquely. For the visually unaffected sample the corresponding numbers of raw reads were 9,452,155, 7,305,262 and 5,634,264, respectively. In this study, we only used reads that aligned uniquely (does not apply to miRDeep2 pipeline). The typical size range corresponding to mature microRNA sequences is between 19 and 25 nt. Among millions of uniquely aligned, high quality reads, 74% and 25% in the visually unaffected and necrotic library respectively, belong to this size range. The visually unaffected sample follows the typical read distribution for small RNA sequencing with a majority of raw reads belonging to the mature miRNA range of 19–25 nt (Figure 1). Both libraries were rather complex in their composition, including various classes of ncRNAs as well as a large number of degradation products of different length originating mostly from coding but also non coding transcripts as well as repetitive elements. The degradation products were particularly distinct in the necrotic sample, which explains the difference in the read size distribution between the two libraries (Figure 1). 

Using reads that aligned uniquely to the pig genome version 9, we found 361,430 read clusters shared between the libraries (Table 1). The number of clusters depends heavily on the cutoff based on the number of reads in the cluster. With cutoffs of 5 and 10, we found 26,465 and 10,331 read clusters, respectively. To reduce the number of false positives on one hand, while preserving the opportunity to discover low expressed miRNAs on the other, we chose to use the 26,465 clusters with raw read count of at least 5 in one of the two libraries for further analysis.

**Table 1:** The result of the alignment, clustering and annotation of the reads

**Classes of reads**	**Raw reads**		**Read clusters**
	**Necrotic**	**Unaffected**	
Raw reads	15034867	12544524	
Filtered reads	11997185	9452155	
Aligned reads	10506718	7305262	
Uniquely aligned reads	7862371	5634264	394631
Clusters with > = 5 reads	7412475	5568204	26465

Clustering of raw reads is obtained after the alignment to the reference genome.

Table 1 shows that in both the necrotic and the visually unaffected samples the number of raw reads forming clusters with more than five reads comprises over 90% (95.7% and 97.6%, respectively) of all the reads.

### Annotation

#### NcRNA detection by homology and class-specific tools

**Figure 2:**
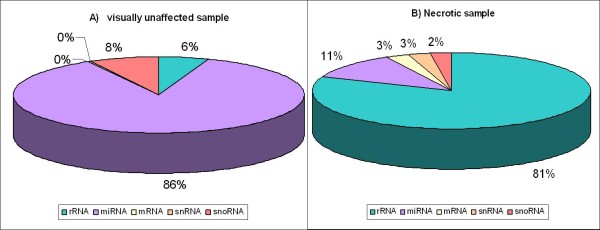
The distribution of raw reads between various RNA classes in A) visually unaffected sample and B) necrotic sample.

We annotated the main classes of ncRNAs using an *in house* ncRNA pipeline (see the Methods section for details) and we used the protein annotation from Ensembl version 56 [33] to annotate the messenger RNAs. Finally, 9,776 out of 24,808 merged read clusters were successfully annotated (Table 2). Various ncRNA classes including miRNA, snRNA, snoRNA, tRNA, scaRNA (169, 40, 169, 73, 11 clusters, respectively) were found. There are great differences in the amount of rRNA/mRNA/microRNA raw reads when comparing the two libraries as shown in Figure 2. This result can be explained by degradation occurring more intensively in the necrotic sample in such an advanced stage of infection. A total of 5,707,949 uniquely mapped raw reads corresponded to rRNA genes in the necrotic sample, while the number of reads annotated as rRNA in the sample from visually unaffected areas was 296,778 reads, or 19 times less. The situation is reversed when looking at the number of reads for microRNAs. The necrotic sample is represented by 783,324 raw reads annotated as microRNA whereas the visually unaffected is represented by 4,652,256 raw reads, which is almost 6 times more than in the necrotic sample (Table 2). This dramatic decrease in miRNA can be understood as follows: As the concentration of rRNAs increases, so does the rRNA sampling probability and the sampling probability of the miRNA must conversely drop. 

**Table 2:** Annotation summary

**Classes of reads**	**Raw reads**		**Read clusters**
	**Necrotic**	**Unaffected**	
Merged clusters with > =5 reads	7412475	5568204	24,808
Un-annotated	317709	135674	15032
Annotated	7094766	5432530	9776
rRNA	5707949	296778	25
miRNA(mirBase hairpin)	783324	4652256	169
Protein	204431	12494	9260
snRNA	178170	2757	40
snoRNA	172592	416729	169
yRNA	29110	18711	5
Conflicts	9511	22853	22
tRNA	6327	6397	73
scaRNA	1801	854	11
Others	1551	2701	3

The read clusters with at least five reads are subjected to annotation. The ncRNAs are annotated with our *in house* ncRNA pipeline; the mRNAs are annotated according to the Ensembl protein annotation of the pig. In case of overlapping annotation the read clusters were merged. The classification of the ncRNAs is in accordance with Rfam. Small Cajal body-specific RNA (scaRNA).

#### Detection of miRNAs by homology

The known miRNAs were identified by BLAST [34] against miRBase or by infernal 1.0 [35] with the models from Rfam9 [36]. We detected 169 miRNAs using high confidence BLAST (95% identity and 95% length) against the hairpins from miRBase version 15. Of the total of 169 miRNAs with annotated hairpins, 142 were *ssc* miRBase hairpins and 27 were annotated hairpins from other organisms. In addition, five miRNAs were not recognized by high confident BLAST, but were matched by Rfam/infernal as members of miRNA families.

#### Detection of novel miRNAs

A distinct pre-miRNA hairpin structure is a primary criterion for microRNA annotation. The hairpin structure and characteristic read patterns of miRNAs makes them much easier to detect than other novel ncRNAs in general [37,38]. In the present study novel miRNAs were predicted with miRDeep2 [39]. The Bowtie [40] mapping performed by the miRDeep2 pipeline reports reads with up to five matches to the genome.

We found 303 miRNAs annotated by miRDeep2 where 221 were from known miRNAs and eight were from other types of ncRNAs. Overlapping the miRDeep2 results with the Novoalign read clusters of at least five uniquely mapped raw reads, we confirm 201 of the 303 miRDeep2 detected miRNAs. Among the 201 clusters found by both miRDeep2 and the clustering of the reads aligned with Novoalign, 4 predictions were also matched to non-miRNA infernal families, 160 were aligned to known miRNAs, and 37 were predicted to be new, un-annotated miRNAs.

These 37 novel predictions were further evaluated by requiring at least five reads detected by miRDeep2, and either both mature and star sequences to be present, or alternatively structure conservation of the site by RNAz pre-release version 2 [41]. After curation, 11 predicted novel miRNAs, which are listed in Table 3, remained. In miRBase 18.0 miR-d7 is annotated as ssc-mir-2320 which was not known in the miRBase version 15 used for the annotation pipeline (Figures showing read profile for each of the novel microRNAs are in Additional files 1 and 2)**.** The Bowtie mapping of the reads revealed a number of known miRNAs not present in the Novoalign mapping of which 24 were expected to be detected only once in the reference genome, but were found twice in close proximity, indicating errors in the assembly ( Additional file 3). 

**Table 3:** The novel miRNAs of the miRDeep2 pipeline

**Novel miRNA (miRBase name)**	**Mature sequence**	**Star sequence**	**Coordinates/strand**	**Raw reads in Necrotic**		**Raw reads in Unaffected**		**Novoalign**	
				**Mature**	**Star**	**Mature**	**Star**	**Necrotic**	**VU**
miR-d1 miR-7142	uuuguuggcuccucugaaguga	acucuccgaggggccuucaaggg	chr2:6684269–6684379:-	30	1	49	0	15	8
miR-d2 miR-7138	gaggacuggccuugcagggugc	ucccagcaaguguccauccaucu	chr2:69969933–69970043:+			21	9	1	4
miR-d3 miR-7137	agcuggucugggaguucccggg	cgggggaacucccagaccagc	chr3:9289209–9289319:+			25	8	5	5
miR-d4 miR-7135	aucugucugugucucugagcag	ucucugagacacugacugugg	chr3-25819302-25819413:+	5	3	72	8	4	14
miR-d5 miR-7134	augcggaaccugcggauacgg	auguccgcggguucccuaucc	chr5:4391270–4391380:-	8,622	32	20,234	161	5413	3690
miR-d6 miR-7139	uagggcacaggaugggaugagg	ccauuccuucgucugugcacuag	chr5:97203430–97203540:+			12	4	2	3
**miR-d7§ miR-2320**	uggcacaggguccagcugucgg	cgaugauggucccuguguuugg	chr6:43886614–43886725:-	28	10	224	37	19	32
miR-d8 miR-7136	ucugguccagacacuguggagc	ucucaguguuugaaccagaagc	chr7:8508420–8508531:-			23	14	3	7
miR-d9 miR-7143	ucugcacuugaagcugagacuga	aagcucagcucugaagugcagagg	chr9:27778763–27778873:+			11	0	1	2
miR-d10 miR-7144	acuuucccgggauuuggagcgc	gcuccuugucccgagaccgcga	chr11:74962849–74962958:+			10	0	11	2
miR-d11 miR-7141	gacgguuuggacguuaagaac	ucuuaacguccaaaccguucc	chr15:129475498–129475608:+			21	0	1	4
miR-d12 miR-7140	aaugaugccccuuagaguugag	caacucaagggggcaucauuca	chr17:37143888–37143999:+	30	1	11	2	14	2

Predictions are confirmed by the unique mappings from Novoalign and either conserved structure according to RNAz or by having reads in both mature and star. 12 miRNAs are novel according to mirBase. Read counts in this table are based on raw reads and refer to the Bowtie mapping used by miRDeep2. VU stands for visually unaffected. **One miRNA (miR-d7) marked with a § is mir-2320, found in mirBase17 so cannot be any longer considered novel.**

### Profiles of expression during APP infection

#### Characterization of the libraries composition

The ncRNAs of the necrotic tissue are dominated by the degraded rRNAs. Other long ncRNAs also show high raw read counts compared to the unaffected tissue. It may, however, be noted that the fraction of tRNAs are comparable in the two samples. The summed raw reads of the microRNAs in particular as well as snoRNAs are much lower in the necrotic sample compared to the visually unaffected one.

We annotated 9260 clusters as being parts of protein coding genes (exons). The raw reads for these clusters are quite different between the samples, 204,431 and 12,494 reads in necrotic and visually unaffected respectively, which underlines the higher degree of degradation of mRNAs in the necrotic tissue which poses challenges in the interpretation of the microRNA expression levels (Table 2).

#### Highly abundant microRNAs

High throughput Illumina GAIIx sequencing revealed a number of highly expressed microRNAs. We present the 20 most abundant microRNAs in both samples (Table 4). Most of the top 20 miRNAs are annotated in pig in miRBase, with the exception of mir-223, mir-144 and a novel miRNA (miR-d5). Noteworthy, the top two, most abundant microRNAs, namely miR-143 and miR-21 are shared between the two libraries. In each sample, miR-143 has by far, the highest number of reads constituting 65% and 49% of all normalized microRNA reads (read counts) in the necrotic and the visually unaffected sample, respectively.

**Table 4:** The top 20 miRNAs in either sample, ordered by number of necrotic reads

**Annotation**	**Annotation coordinates**	**Normalized read counts**		**log2(FC)**	**p-value**
		**Necrotic**	**Unaffected**		
mir-143	chr2:136030751–136030831:+	697353.81	461481.09	−0.60	3.64E-001
mir-21	chr12:34201564–34201656:+	57776.26	122252.79	1.08	1.02E-001
mir-451	chr12:42820765–42820830:-	49029.44	3944.06	−3.64	5.16E-007
mir-30a	chr1:53848997–53849104:-	46416.19	109713.26	1.24	6.12E-002
mir-148a	chr18:45214547–45214615:-	25801.77	26340.33	0.03	9.64E-001
let-7 g	chr13:28999418–28999498:+	23644.76	10926.89	−1.11	9.22E-002
mir-126	chr1:294113068–294113141:+	15131.46	54694.16	1.85	5.98E-003
mir-30e	chr6:121824300–121824380:-	12711.24	15622.78	0.30	6.49E-001
mir-142	chr12:32630402–32630482:-	11700.22	2961.25	−1.98	3.43E-03
miR-d5	chr5:4391269–4391380:-	10960.52	3887.77	−1.50	2.51E-02
miR-223	chrX:51247504–51247526:-	10712.16	367.80	−4.86	2.38E-10
mir-144	chr12:42820909–42820993:-	8968.19	1426.54	−2.65	1.34E-04
mir-10a	chr12:22389423–22389503:-	8433.66	26108.75	1.63	1.50E-02
mir-30d	chr4:5729458–5729537:+	8170.45	10106.35	0.31	6.39E-01
mir-23a	chr2:56861158–56861228:-	5968.90	3586.67	−0.73	2.64E-01
mir-27b	chr10:26148775–26148855:-	5775.87	10319.10	0.84	2.03E-01
mir-191	chr13:26605640–26605720:-	4910.64	3262.54	−0.59	3.69E-01
mir-146b	chr14:118471587–118471686:+	3848.33	4350.12	0.18	7.87E-01
let-7c	chr13:130665020–130665114:+	3605.36	5812.52	0.69	2.94E-01
mir-146a	chr16:60604389–60604467:+	3486.58	635.24	−2.46	3.68E-04
mir-23b	chr10:26149009–26149089:-	2741.48	3640.56	0.41	5.33E-01
mir-1	chr17:64096221–64096329:+	1986.93	3300.20	0.73	2.66E-01
mir-152	chr12:21743214–21743294:+	1764.21	3394.96	0.94	1.53E-01
mir-99b	chr6:39533215–39533285:+	1434.86	3234.90	1.17	7.72E-02

The reads shown in the table are normalized to counts per million calculated by the TMM normalized sample size, the log2 of the fold change (positive for down regulation in the necrotic tissue) and the p-values are determined by the exact-test.

After normalization of the raw reads accordingly we found that 17 of the 180 known and novel miRNAs were significantly up regulated in the necrotic sample with an e-value cutoff of 0.05. Similarly, 12 miRNAs were significantly down regulated (Table 5). Interestingly, the highly expressed novel miRNA on the negative strand of chromosome 5 called miR-d5 is present in the top 20 most abundant microRNAs and shows up regulation in the necrotic sample (Table 4 and Table 5). This novel microRNA is only found in pig, cow and dolphin. The complete list of 180 known and novel miRNAs is provided in Additional file 4.

**Table 5:** The miRNAs significantly up or down regulated in the necrotic tissue, ordered by the fold change (FC)

**Annotation**	**Annotation coordinates**	**Normalized read counts**		**log2(FC)**	**p-value**
		**Necrotic**	**Unaffected**		
miR-223(Q)	chrX:51247504–51247526:-	10,712.16	367.80	−4.86	2.38E-10
miR-d12	chr17:37143883–37144004:+	28.35	1.80	−3.97	2.65E-05
mir-451(Q)	chr12:42820765–42820830:-	49,029.44	3,944.06	−3.64	5.16E-07
mir-582	chr16:36589520–36589618:+	193.02	25.64	−2.91	6.40E-05
mir-7	chr1:200316408–200316468:-	708.65	111.38	−2.67	1.37E-04
mir-144(Q)	chr12:42820909–42820993:-	8,968.19	1,426.54	−2.65	1.34E-04
mir-146a(Q)	chr16:60604389–60604467:+	3,486.58	635.24	−2.46	3.68E-04
mir-15b	chr13:78787510–78787608:+	1,150.04	278.66	−2.05	2.70E-03
mir-19a	chr11:60972740–60972822:+	114.73	28.65	−2.00	5.07E-03
mir-142(Q)	chr12:32630402–32630482:-	11,700.22	2,961.25	−1.98	3.43E-03
miR-d1	chr2:6684268–6684379:-	31.05	8.21	−1.92	1.83E-02
mir-132	chr12:45599471–45599572:-	82.34	25.04	−1.72	1.70E-02
mir-222	chrX:40478833–40478913:-	134.98	42.87	−1.65	1.77E-02
mir-221	chrX:40478093–40478163:-	827.44	271.44	−1.61	1.71E-02
miR-d5(Q)	chr5:4391269–4391380:-	10,960.52	3,887.77	−1.50	2.51E-02
mir-505	chrX:112590811–112590891:+	62.09	22.04	−1.49	3.84E-02
mir-128-1	chr15:14550873–14550955:-	107.99	40.47	−1.42	4.40E-02
mir-542	chrX:108481538–108481618:-	715.40	1,961.41	1.46	2.97E-02
mir-10a	chr12:22389423–22389503:-	8,433.66	26,108.75	1.63	1.50E-02
mir-30c-2	chr1:53819905–53819985:-	80.99	257.42	1.67	1.63E-02
mir-181a-1	chr10:22839351–22839455:-	37.79	122.60	1.70	1.90E-02
mir-138a	chr13:23336889–23336951:+	82.34	272.45	1.73	1.31E-02
mir-497	chr12:49575593–49575673:+	51.29	181.10	1.82	1.03E-02
mir-126(Q)	chr1:294113068–294113141:+	15,131.46	54,694.16	1.85	5.98E-03
mir-450b	chrX:108480528–108480608:-	66.14	248.61	1.91	6.66E-03
mir-100	chr9:47702720–47702800:-	527.78	2,599.26	2.30	8.24E-04
mir-181b-2	chr1:280335867–280335953:+	2.70	17.03	2.66	2.02E-02
mir-450a	chrX:108480687–108480793:-	0.00	13.62	Inf.	1.14E-03
mir-326	chr9:9132229–9132320:-	0.00	5.01	Inf.	4.00E-02

The reads shown in the table are normalized to counts per million calculated by the TMM normalized sample size, the log2 of the fold change (positive for down regulation in the necrotic tissue) and the p-values are determined by the exact-test. A p-value cutoff of 0.05 was applied. The miRNAs marked with (Q) were chosen for RT-qPCR.

The top 20 most expressed snoRNAs show a much larger variation between the two samples. Nine are common in the top 20 list of most expressed snoRNAs in the two respective samples (for details see Additional files 5 and 6). The present study however, was mainly focused on microRNA expression therefore snoRNAs are not investigated in details.

**Figure 3:**
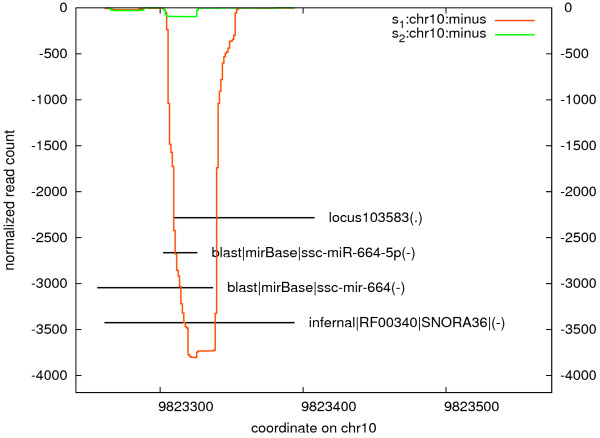
**Profile of reads distribution for read cluster annotated as both miR-664-5p and SNORA36.** S1-necrotic, S2-visually unaffected sample.

The high throughput sequencing results were further subjected to validation of candidate miRNA expression by RT-qPCR. Most of the miRNA candidates are falling into highly abundant or up/down regulated microRNAs in the necrotic sample, however the two miRNAs: miR-15a and miR-155 were chosen due to their biological relevance as reported in the literature disregarding their rather low read count ( Additional file 4). The SNORD15 is the third highest expressed snoRNAs, up-regulated in the necrotic sample ( Additional file 5). The ncRNAs chosen for RT-qPCR validation were: miR-15a, miR-21, miR-126, miR-142-5p, miR-143-3p, miR-144*, miR-146a-5p, miR-148a, miR-155, miR-223, miR-451, miR-664-5p, miR-d5 and SNORD15. Ten of the candidates were highly expressed in both samples (Table 4) of which seven were found differentially expressed by RNAseq (Table 5). miR-664-5p is an interesting case of a so called snoR-like microRNA [42]. The annotation procedure assigns both miR-664-5p and SNORD36 to the same locus. For this reason miR-664-5p is neither featured among microRNAs nor among snoRNAs (Figure 3). 

#### qPCR validation of candidate microRNA expression

High throughput data demand validation of particular candidates by a technique with higher specificity and sensitivity like RT-qPCR. In this study, SYBR Green RT-qPCR was used to verify the expression of 13 select microRNAs and one snoRNA. Within 13 microRNAs, 12 are annotated in miRBase and one is a novel microRNA discovered in the present study. Additionally, miR-152 and miR-191 were used as RT-qPCR reference genes.

**Figure 4:**
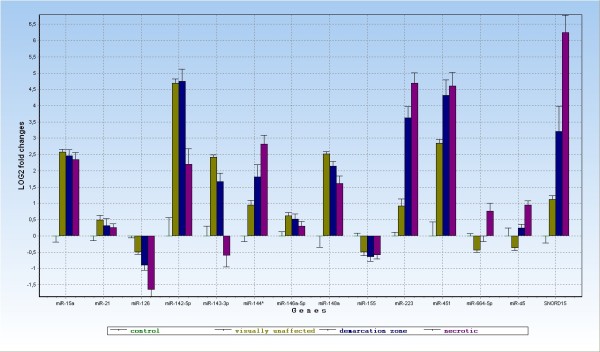
**RT-qPCR analysis of expression of 13 selected unique miRNAs and one snoRNA.** miR-d5 represents a novel unannotated microRNA. Data presented are normalized to the reference genes, LOG2-transformed relative quantities. The level of expression for control sample is set to zero.

In addition to RNA isolated from visually unaffected and necrotic tissues, RNA from the demarcation zone (between infected and non-infected tissue), and from nose and trachea originating from the same infection study were included in the RT-qPCR studies. Moreover, developmental lung (gestation day 50, gestation day 100 and 3 months old ( adult control) were included in order to obtain microRNA expression profiles across tissues present in the respiratory tract as well as across developing uninfected lung tissue. The data for trachea, nose, F50, and F100 are not included in the main manuscript however the diagram including all the tissues is present in Additional file 7. To simplify the graphical representation of microRNA expression profiles we have included results from adult (not infected) control lung, necrotic area, demarcation zone and visually unaffected area only (Figure 4). For data representation, expression in the control sample was set to zero.

**Figure 5:**
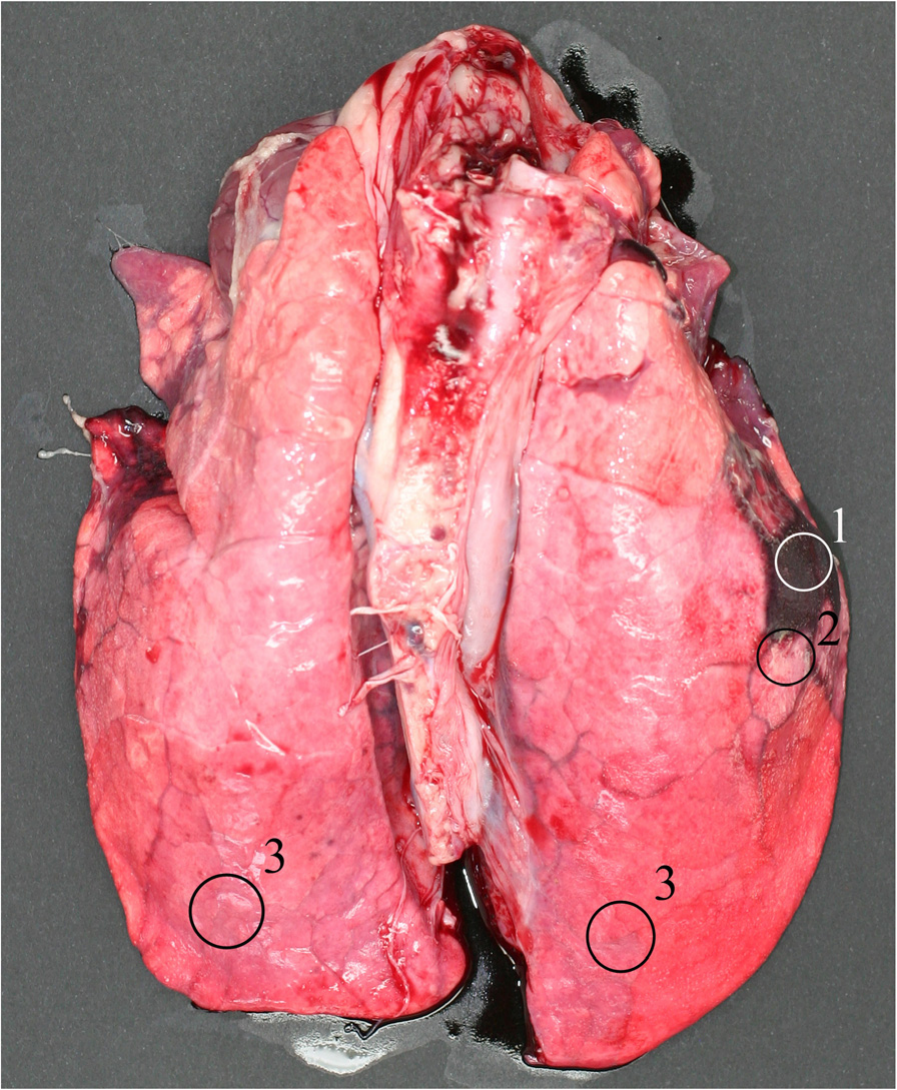
**Sampling areas of the porcine lungs infected with *Actinnobacillus pleuropneumoniae*.** 1 – Necrotic area, within pulmonary lesion. 2 – Demarcation zone, border area between visually unaffected and pulmonary lesions. 3 – Visually unaffected area, which was sampled either from unaffected lung lobe (3 to the left) or from site as distant from pulmonary lesion as possible (3 to the right).

The ANOVA performed on LOG2 transformed relative quantities revealed significant differences in the ncRNA expression between the analyzed tissues. As mentioned above, Illumina GAIIx high throughput sequencing was performed on pools of visually unaffected and necrotic sample only. RT-qPCR, on the other hand provides additional, complementing results for the demarcation zone as well as a control sample group. Taking the nature of the demarcation zone sample (tissue sampled at the border of necrotic and visually unaffected tissue, see Figure 5) we would expect the intermediate microRNA expression rates. The expression levels of all assayed microRNAs but one and of the snoRNA in the demarcation zone sample are intermediate in relation to the visually unaffected and necrotic areas.

We arranged the expression profiles of the genes selected for RT-qPCR in five groups.

Group 1: microRNAs up-regulated in all or some of the infected samples compared to control (miR-144* (miR-144-5p in the newest miRBase 18.0 in human), miR-223, miR-451, miR-664-5p, miR-d5, SNORD15), (p-values shown in Additional file 8). All of the above microRNAs and snoRNA show significant up-regulation of expression in necrotic vs. visually unaffected sample. Such level of relative expression (RT-qPCR) is highly comparable to number of reads (sequencing) for each of the above six ncRNAs.

Group 2: microRNAs down-regulated in necrotic comparing to visually unaffected areas and/or control sample (miR-126, miR-155). The read counts for miR-126 also showed significantly lower number of counts for the necrotic sample in comparison to the unaffected sample. MiR-126 has the highest expression in control sample, which decreases significantly with the necrosis progression. MiR-155 has a slightly different profile with all the infected samples having significantly lower expression than the control sample but at the same time the expression does not differ significantly between samples representing various stages of infection (similar read count number confirms this expression pattern).

Group 3: MicroRNAs down-regulated in control followed by up-regulation in visually unaffected and down-regulation again in necrotic sample (miR-142-5p, miR-143-3p, miR-148a). Despite the decreasing trend, the expression for miR-142-5p and miR-148a, in the necrotic sample remains significantly higher than in the control. In contrary, miR-143-3p makes a slight exception by having the expression in necrotic area down-regulated significantly compared to the control. Expression profile of miR-142-5p similarly shows lower read count number in visually unaffected vs. necrotic sample obtained by sequencing. Surprisingly the differential expression detected by RT-qPCR does not confirm the lack of significant differences for the miR-148a and miR-143 shown by RNAseq data.

Group 4: microRNA highly up-regulated in infected samples vs. control, showing no differences between the different sampled areas from infected lung (miR-15a). Among all unique qPCR candidates, miR-15a represents an exclusive profile of very low expression in the control sample, followed by remarkable up-regulation (p-value =1 E-08) in all 3 samples originating from the infection study. No significant difference of the miR-15a expression has been detected between the infected samples (visually unaffected, demarcation zone, necrotic), which is also reflected in the RNAseq results.

Group 5: Two microRNAs showed no significant difference (miR-21) between the investigated tissues or a slightly significant difference (miR-146a-5p) between control and visually unaffected area (p-value = 0,48). In contrary, the RNAseq results show up-regulation of miR-146a-5p placing it within 20 most regulated microRNAs in necrotic sample. MiR-21 is by far, the most expressed of all the assayed microRNAs, showing very high, stable expression in the lung tissue, regardless the presence/degree or absence of infection. However, according to read counts miR-21 is placed as the second most abundant transcript, right after miR-143. Furthermore, the relative expression of miR-21 detected by RNAseq points towards up-regulation of this microRNAs in visually unaffected sample in comparison to the necrotic sample (visually unaffected sample contains more than 2 times more reads than the necrotic).

### Target predictions of microRNAs

Regardless of the limited understanding of microRNA function, microRNA target predictions point out mRNAs possibly regulated by individual miRNAs. We combined (conservative) human data from four of the prominent target prediction methods sources namely: Targetscan [43-46], pictar [47,48], miRanda [49,50] and microT [51]. We only included conserved miRNAs and conserved targets. The merged data contained 1,365,255 predicted interactions between 18,542 proteins and 789 miRNAs. A total of 272,641 predicted protein/miRNA targets were in agreement with two or more of the four methods and these constituted our conservative set. For further miRNA target investigation, the range of possible targets of interest was narrowed down to 91 transcripts coding for proteins based on the available literature regarding microRNAs in bacterial infection, immunological response and cell death [14], [15] as well as the transcriptional profiling performed at different sites of lungs in pigs during acute bacterial respiratory infection [23], [52]. Moreover, the set of microRNAs subjected to mRNA predictions was limited to 12 microRNA candidates validated by RT-qPCR (excluding novel miR-d5 and SNORD15). These 12 microRNAs are all conserved in human as are the proteins targeted by them and we therefore assume that the microRNA-protein interaction is conserved as well.

Overlap of the mRNA and miRNA predictions resulted in 149 predicted interactions for the 91 proteins with the 12 miRNAs of which 35 interactions (corresponding to 26 distinct proteins) are predicted by two or more methods and are shown in Additional file 9.

The miRNA candidates assayed by RT-qPCR are found to target multiple mRNAs as shown in Table 6. Two out of 13 microRNAs (miR-15a, miR-155) included in Table 6 are predicted to target more than 20 different mRNAs. The following five miRNAs (miR-21, miR-143-3p, miR-146a-5p, miR-223, miR-664-5p), target over ten different protein coding genes. MiR-142-5p, miR-148a and miR-451 target over five mRNAs, whereas only two predictions were found for miR-126.

**Table 6:** Predicted mRNA targets for unique miRNA candidates validated by RT-qPCR

**Annotation**	**gene targets**
miR-15a	ALB, BCLAF1, C1QA, C1QB, CD163**, CYP19A1, CYP26B1**, FKBP1A**, FOXP3, IFNGR1, IL10RA**, IL10RB, IL18R1, IL6R, IRAK1BP1, MAPK8, NFKB1, PAK2, PDCD4**, SFTPD, SMAD7**, SOCS5**, TAB3**, TNFAIP1**
miR-21	CYP19A1, IL10, IL12A**, IL1B**, IRAK1BP1, PDCD4**, SFTPA1, SMAD7**, SOCS5, TAB2, TAB3**, TNFAIP3, ZNF41
miR-126	IL21R, TOM1**
miR-142-5p	BCLAF1, CYP26B1, FKBP1A**, IL21R, IL6ST, MCL1**, SMAD2, TNFAIP3, TNFAIP6
miR-143-3p	ALB, FN1, IFNG, IL10RA, IL10RB, IL18R1, IL21R, IL6R, IL6ST, IRAK1BP1, KHSRP, MAPK8, PAK2, POSTN TAB2, TNFAIP1, ZEB2, ZNF41
miR-146a-5p	**BCLAF1**, CCBP2**, CCL5, FN1, FTH1, HLA-A, IL6, IRAK1**, POSTN, SMG1, TAB2, TERF1, TNFAIP3, TNFAIP8, TRAF6**, ZEB2, ZNF41
miR-148a	ALB, C8A, CYP26B1, HLA-A, IL6ST, IRAK1, SMAD2**, SMAD7, ZEB2
miR-155	BCLAF1, CCL5, CYP26B1**, FN1, FTH1, IFNG, IL12A, IL13**, IL18R1, IL6ST, ILF3, INPP5D**, IRAK1BP1, PAK2**, PDCD4, SMAD2**, SOCS5**, TAB2**, TERF1**, TNFAIP6, TNFAIP8, ZEB2
miR-223	BCLAF, C8A, CYP26B1, IL6ST**, IRAK1BP1, KHSRP, SMG1, SOCS5, TAB2**, TNFAIP8, ZEB2, ZNF41
miR-451	CFI, IL6, IL6R, PAK2, TNFSF15, TOM1
miR-664-5p	A2M, CYP26B1, IFNG, IL18R1**, IL1A**, IL8, JUN, KHSRP, MAPK8, MCL1, NFKB1, SMG1**, TERF1, TNFAIP1**
miR-d5	No predictions found

Only widely conserved target sites included. ** indicate target predicted by two or more algorithms. Bold and underlined protein coding gene symbol indicate miRNA:mRNA interaction featured in StarBase which includes CLIP-Seq experimental validation.

The target predictions for the 11 novel miRNAs were performed with TargetScan against human 3’ UTRs, keeping only the widely conserved target sites. For the novel miRNAs we found a total of 1,926 targets. Only eight out of 1,926 predicted interactions target mRNA within the selected 91 proteins and these are: CYP26B1: miR-d11, ILF3: miR-d10, MCL1: miR-d7, PAK2: miR-d3, miR-d7, miR-d8, SMAD7: miR-d12. SOCS5: miR-d7. The highly expressed novel miRNA miR-d5 was found to target only five transcripts, which is an unusual low number compared to the others. This characteristic is shared with miR-d11 and it is likely because both miRNAs are linage specific and not found in human (see Additional file 10) which also explains why their targets are missing from the set of widely conserved target sites. All targets without consideration of conservation of the target sites are given in Additional file 11, however, since the pig is not part of the TargetScan data set while cow is, we chose to use the cow 3’ UTRs. Thus, we found no targets of particular interest mostly due to only partial sequence conservation of both miR-d5 and miR-d11between pig and cow.

## Discussion

In the present study we have mainly been interested in investigation of microRNAs involved in the progression of the APP infection rather than comparing infected versus healthy individuals. Furthermore, we decided to use infected and visually uninfected tissue from pigs infected with the bacterial pathogen *Actinobacillus Pleuropneumoniae* in order to minimize the genetic and phenotypic diversity between samples. We have used these samples to generate expression profiles of microRNA by high throughput. Working with infected tissue, which shows severe signs of advanced necrosis, is challenging because RNA degradation has to be taken into account. As expected, the annotation of the RNAseq data revealed a dominating presence of degradation products in the necrotic sample, regardless of the fact that all the samples included in the present study showed RNA quality indexes acceptable for gene expression studies (RQI > 6.0). The read length distribution in the visually unaffected sample is comparable to profiles generated in other studies [53], [54] with the highest number of reads belonging to the 19-25nt microRNA range which suggests the reliability of the library construction and the sequencing. A meaningful expression profile can only be generated if the raw reads are normalized in accordance to the degree of degradation. To avoid letting rRNA and mRNA degradation products over-shadow the read counts of the miRNAs among the reads generated in the necrotic sample, the miRNAs from both samples were studied separately. Two different mapping methods have been used in this study: one with unique reads using Novoalign [31], and one with matches to maximum five genomic loci using Bowtie. The results generated by the two methods are in strong agreement with each other.

The sequencing data provide evidence of microRNA deregulation during lung infection with the bacterial pathogen. Both up- and down-regulation of microRNAs were found when comparing the expression in the necrotic versus visually unaffected tissue of the lung. A subset of the highly expressed and biologically relevant genes (13 miRNAs and one snoRNA) was subjected to RT-qPCR. In 10 out of 14 genes, RT-qPCR results supported the results obtained by deep-sequencing indicating that the data generated reflects a biologically meaningful profile. The disagreement featured in the remaining four microRNAs is most likely pointing towards sequencing bias accompanying RNAseq experiments rather than misevaluation of the expression by a specific and sensitive RT-qPCR.

The number of annotated microRNAs in domestic pig in the newest miRBase 18.0 is still much smaller than in other organisms, featuring 1527 entries for human, 741 for mouse and only 228 for pig. Multiple alignments to the pig genome and 17 other organisms were performed based on the assumption that the majority of miRNA sequences are conserved among species [2], [3]. A total of 27 of the annotated 168 microRNAs matched hairpins annotated for other organisms than pig in miRBase and based on sequence homology they could be annotated in pig enriching the repertoire of porcine microRNAs by 27 homologous miRNAs. Furthermore, 11 novel microRNAs were found by search performed with miRDeep2 pipeline [39][55]. Often, novel microRNAs are not represented by many read counts. An exception to that rule is one of the detected miRNAs, annotated at the negative strand of chromosome 5, called – miR-d5, which is represented by 5413 and 3690 read counts in necrotic and visually unaffected sample, respectively. Moreover, this novel microRNA was found in only two other organisms: cow and dolphin out of the 17 different reference genomes tested.

Studies on high throughput sequencing of small RNAs show that there are in most cases a few distinct microRNAs that constitute the majority of microRNA reads [56]. Our study also reports highly abundant microRNA namely miR-143 which is represented by 65% and 49% of the miRNA reads in the visually unaffected and the necrotic sample, respectively. RT-qPCR validation of this microRNA did, not reflect such a high level of expression. Even more striking is the fact that this particular microRNA has not previously been described as extremely abundant in mammalian lung, neither has it been pointed out as a major regulator of immune response [57]. A possible explanation for the high read count in both libraries could be that during the library construction an artifact has been introduced in the RNAseq experiment.

The second most abundant microRNA in both samples – miR-21 is involved in many biological scenarios [58-61]. In the present study, miR-21 shows down-regulation in the necrotic sample in comparison to the visually unaffected one, however supported by quite low p-values (Table 4). RT-qPCR performed on miR-21 detected the highest expression level of all the assayed microRNAs. No differential expression was found between the samples included in the RT-qPCR experiment, which could indicate an important role of miR-21 in the homeostasis of lung.

The 12 microRNA candidates assayed by RT-qPCR were investigated for target predictions in order to provide more insight into the biological pathways where the different microRNAs could play a role. The conservative predictions indeed anticipated a number of very promising, biologically interesting interactions. One of the major factors involved in detection of pathogens and initiation of inflammatory response are TLRs. Moreover, a rapid immune response is triggered upon pathogen invasion by the presence of LPSs – a major component of the gram negative APP membranes. After binding of LPS to the TLR4/MD2/CD14 receptor complex, signalling pathways lead to the activation of nuclear factor κB (NF-κB) that further regulates a large set of genes critical to cell proliferation, inflammation and innate immunity [62]. Literature reports a number of microRNAs, mainly miR-21, miR-155, miR-146a, miR-223 to be important regulators of the protein coding genes involved in the above mentioned pathways [60], [61], [63]. Studies on microRNA expression in bacterial infections of various sources similarly point towards the same microRNAs [20], [17], [64], [65] as the key players in the host response. We found 17 microRNAs up regulated in necrotic versus visually unaffected sample, while the protein coding study on the same biological material found that the highest number of deregulated genes was in the necrotic area [23]. The microRNAs showing remarkably high expression level in our study namely miR-21 is a powerful anti-inflammatory regulator, which by direct targeting of a PDCD4 gene inhibits the pro-inflammatory regulator NF-kB. Moreover, the levels of anti-inflammatory cytokine IL-10 are increased upon miR-21 up-regulation [65]. However, our study detected an extremely high expression of miR-21 in all the samples, regardless the infection status. Similar results of high expression of miR-21 have been found in viral infection of porcine dendritic cells [66].

Two targets of miR-21: Interleukin I Beta (IL1β) and tumor necrosis factor, alpha-induced protein 3 (TNFAIP3), which is rapidly induced by the TNF were assayed by RT-qPCR on mRNA in another APP infection study in pigs [23]. In the study of Mortensen and collaborators IL1β was up-regulated in infected samples and TNFAIP3 was repressed. Ideally, we would expect an up-regulation of microRNA while mRNA is attenuated and vice versa. However, the lack of such correlation is not necessarily contradictive to the nature of microRNA mediated regulation. A single mRNA can be fine-tuned by multiple microRNAs and this regulation is a very complex, multi-factorial and time dependent network and possibly samples taken at different time points of infection would result in slightly different profiles.

The following key player in control of immunity and inflammation, miR-155, showed rather contradicting results to those existing in the literature [18]; [67]; [68]. In our RT-qPCR study, miR-155 was significantly down-regulated under pathogen triggered inflammation and so was mRNA coding for the miR-155 target IFN-γ in the same animals [23].

The 5b APP serotype used in the present study is highly virulent. It has been show that the lethality of pleuropneumonia in pigs is not necessarily caused by ventilator failure [69] but by a septic shock-like condition, which develops when, immunological and inflammatory responses are unbalanced and uncontrolled release of cytokines occurs [8]. Such dramatic disturbance in regulation of immune response could be the explanation for the surprisingly reversed miR-155 expression profile.

Another microRNA candidate namely miR-223 was investigated in the present study. We indeed detected a remarkable, gradually increasing expression of miR-223 with infection progression, in infected samples in comparison to the control. Moreover, Matsushima and co-workers (2011) found miR-223 to be the only up-regulated microRNA in *Helicobacter pylori*-infected gastric mucosa. TLR4 and TLR3 pathways are possibly targeted by miR-223 [64].

Nearly all up-regulated microRNAs in lung tissue from infected pigs target IL-6R and/or IL6ST, in addition miR-144 and miR-146a 5p targets mRNA coding for IL-6. IL-6 was found to be highly up-regulated in the same animals [24] 14–18 h after experimental infection. We might speculate that expression of this pro-inflammatory cytokine as well as its receptor will be tightly regulated in order to avoid host mediated tissue damage. Unpublished time-course studies of mRNA from lung tissue reveal IL-6 first to be highly up-regulated and then to be significant down-regulated somewhere between 12 and 24 h after infection with APP (Skovgaard, personal communication) MicroRNA miR-148a and miR-126 are also mentioned in the inflammation related literature with miR-148a having implication in function of primary bronchial epithelial cells [70] and miR-126 in chronic asthma where initial increase in expression of this microRNA was found [71]. The miR-148a similarly to miR-146a-5p was found to target mRNA encoding IRAK1. Our most confident prediction for miR-148a is a SMAD2 gene involved in regulation of cell growth and proliferation - two processes which are rather silenced during stressful bacterial infection. This corresponds to great up-regulation of miR-148a in infected tissue. Interestingly, Interleukin 21 receptor (IL21R) is regulated by miR-126. Interleukin 21 is yet another immunoregulatory cytokine though to be involved in transition from innate to adaptive immunity. The remaining microRNA candidates namely: miR-15a, -142-5p, -144, -451 and −664 were not previously described as important in response to infectious pathogen lung infection. Nevertheless, we provide a very distinct and significant expression profiles supported by multiple target predictions for those microRNAs, which suggest that they might be a new coming group of microRNAs involved in inflammation regulatory networks.

## Conclusion

This is to our knowledge the first study to survey the host microRNA expression profiles in *Actinobacillus pleuropneumoniae* lung infection in pig. We provide insight into involvement of a collection of microRNAs in regulation of immune and inflammatory response during porcine lung infection. A number of those microRNAs have not previously been described as regulators of immune system, which might point towards their possible organ specific, infection time specific or species specific role. Furthermore, the atlas of annotated and novel porcine microRNAs expressed in different areas of infected lungs is described. The complexity of the microRNA-target regulation suggests that the network should be rather viewed as a multi-factorial structure that fine-tunes plasticity of inflammation rather than a set of isolated regulatory events. We believe that better understanding of the possible role of microRNAs modulating PRR signaling in response to pathogens, by targeting different components of these complex pathways firstly contributes to better understanding of the complex regulation and secondly may contribute to drug improvements in the future. This study builds a background for miRNA-target interaction based-research in APP infection in pig. Furthermore, the findings of the present study combined with translational studies can contribute to elucidate mechanisms responsible for susceptibility to and pathophysiology of lung infection in other organisms including humans.

## Methods

### Experimental infection

Ten, 8–10 weeks old castrates, Danish crosses between Landrace/Yorkshire/Duroc, free from *Actinobacillus pleuropnemonia*, were inoculated intranasally with *A. pleuropneumoniae* serotype 5b, isolate L20 as described previously in [72]. Re-isolation of the inoculum strain of *A. pleuropneumoniae* was performed from all inoculated animals. Severity of lung lesions characteristic of pleuropneumonia was rated by a highly skilled pathologist. Animals were sacrificed 14–18 h after the inoculation. All animal procedures were approved by the Danish Animal Experiments Inspectorate. Lung, trachea and nose tissues were sampled and immediately snap frozen in liquid nitrogen and stored in −80 °C until used. Lung samples were taken from three different sites: necrotic area, demarcation zone, and visually unaffected area. For detailed description of the infection study see [23]. For present study, samples of necrotic lung of eight animals (severe lesions and necrosis present in the lung tissue), of demarcation zone in lung of eight animal (sample taken at the border area between visually unaffected and pulmonary lesions) and samples of visually unaffected lung from ten animals (healthy-looking unaffected lung tissue) (Figure 5), trachea and nose (ten animals each) were taken. Furthermore, additional samples were obtained from control porcine lung tissue (control samples independent of the infection study), porcine lung tissue from fetus gestation day 50 (F50) and porcine lung tissue from fetus gestation day 100 (F100). All three of the above groups were free from APP infection.

### RNA extraction

Total RNA was extracted from each necrotic lung, visually unaffected lung, demarcation zone lung, trachea, nose, F50, F100 and control porcine sample, using Tri Reagent® (Molecular Research Center, Inc., USA) following the manufacturers recommendations. App. 300 mg of each tissue was used for the isolation procedure. RNA quantity was determined on a Nanodrop 1000 (Peqlab Biotechnologie, Germany). Additionally, the integrity of the total RNA samples was measured by total RNA Assay and Experion RNA StdSens Analysis Kit using 2100 Bioanalyzer (Agilent Technologies, Santa Clara, CA, USA), and Experion (Bio-Rad Laboratories, Inc., Hercules, CA, USA) respectively. The RNA concentrations assessed by Nanodrop as well as the integrity values of RIN and RQI are provided in the Additional file 12.

### Small RNA library and Solexa sequencing

Total RNA fractions from eight necrotic and ten visually unaffected samples were pooled, respectively and used for the small RNA library construction following standard Illumina protocols version 1.5. Briefly, RNAs were ligated to a 5’ and a 3’ adapter sequentially, reverse transcribed into cDNA, PCR amplified with adapter specific primers, and finally the small RNAs were purified on 3% MetaPhor® Agarose gel (Cambrex Bio Science Rockland, USA) to generate suitable length of tags for small RNA sequencing performed on Illumina Genome Analyzer IIx (Illumina GAIIx).

### Computational analysis of high throughput sequencing data

#### Alignment and clustering of reads

The reads were filtered for chastity by the Illumina pipeline, after which they were aligned to version 9.0 of the pig genome using Novoalign version 2.05.25. The reads were stripped of adapter allowing for up to two mismatches, and minimum insert lengths of 18 nucleotides were required prior to alignment. Novoalign's microRNA scoring scheme was employed. All alignments, no more than five points away from the best alignment are reported by Novoalign. Novoalign filters for quality, strips adapter sequence, and filters out homo-polymer reads prior to aligning to the genome. Only the aligned reads mapping uniquely were used for read clustering. The reads in each library were clustered allowing for a gap of up to 15 uncovered nucleotides before breaking a cluster. Clusters were then merged between the two libraries to get one consistent set of clusters.

#### Annotation

The read clusters were annotated using a number of methods: The Ensembl protein annotation for build 9.0 of porcine genome; the high confidence BLAST (95% id, 95% length) [34] against Rfam 9.1 [36] the snoRNA database version 3 [73], the tRNA database [74], the miRBase version 14 (miRBase version 16 for porcine miRNAs) [75]; the RNAmmer version 1.2 for ribosomal RNAs [76]; the tRNAscan-SE for tRNAs [77]; and finally Infernal 1.0 [35]. Rfam 9.1 against all Rfam models (the results where filtered with the model specific gathering score plus an infernal e-value cutoff of 1e-3). BLAST conflicts were resolved by taking the ncRNA that scored best e-values. When a cluster was annotated by more than one method, the conflicts were checked manually. Annotation is strand specific and is further cleaned according to the number of reads covered on a given strand. Annotations, which accounted for less than 40% of the reads on the same strand in a cluster were dropped. If an annotation spanned over more than one cluster, the clusters were merged prior to further analysis.

#### Cross species conservation and novel ncRNAs

A UCSC (The University of California, Santa Cruz) genome browser style multiple alignment based on pig and 17 other vertebrates was performed using lastz [78] and the UCSC tool chain as part of our *in house* ncRNA pipeline. Pairwise alignments on chunked up genomes were performed by lastz, followed by chained alignments, and single best coverage alignments (nets) of the target genome (pig) with the UCSC tool chain. The single best coverage alignments were cleaned up by synteny when the query genome assembly was chromosome based and by reciprocal best alignments between query and target when the query genome assembly was scaffold based. Pig centered, multiple alignments were formed from the pairwise alignments with the *roast* program from the tba/multiz [79] package using a minimal alignment block size of 40. Structured novel ncRNAs were predicted from the multiple alignments by RNAz version 2 pre-release based on structure conservation in between species [80].

#### Search for novel miRNAs

Novel miRNAs were explored using the miRDeep2 pipeline [39]. The reads were aligned using Bowtie and reads matching up to five different places in the genome were used for further analysis. A maximum of 1 mismatch was allowed. The aligned reads were checked for known miRNAs using the mature miRNAs from *Sus scrofa, Homo sapiens, Equus caballus, Bos Taurus,* respectively and the full hairpins from *Sus scrofa*, all from miRBase version 17. The miRDeep2 pipeline confirms the folding ability of the miRNAs with RNAfold, and the miRNA probability is checked with Randfold. We further restricted the set of novel miRNAs by confirming them with the unique mappings of the Novoalign alignment and by requiring either structure conservation by RNAz or by confirming both mature and star sequence reads.

#### Normalization

In this experiment the libraries and raw reads could not be compared directly due to large variation in the fractions of degraded RNAs. We therefore chose to treat the miRNAs from each sample separately, which leads to summed miRNA raw reads of 791541 and 4672073, respectively for the 180 miRNAs detected in the samples. When the samples as here are very diverse it is further recommended to calculate normalization factor by the threaded means of M-values (TMM) method [81]. We found normalization factors of 0.9359472 and 1.0684363, respectively. The p-values of the relative expressions in the two tissues were calculated by the exact-test build into the edgeR package [82]. An estimation of sample dispersion is needed to use this test, and in the absence of replicated samples we follow the authors’ recommendation of using a dispersion factor of 0.1 for genetically identical samples.

#### Target predictions

We used miRNA/protein target data for human from the following sources TargetScan [43-46], PicTar [47], [48], miRanda [49], [50] and microT [51]. TargetScan version 5.0 vertebrate dataset with conserved target sites was used. For PicTar we used the four way results for hg17 downloadable from the UCSC browser; the august 2010 dataset with “good scores” and conserved target sites were used for miRanda. For microT, the version 4 dataset with a score cutoff of 0.3 was applied. The protein annotations were transferred to Ensembl gene identifiers and from there to gene symbols using biomart [83]. Some protein annotations did not have matching identifiers or gene symbols therefore were discarded. The predictions for the novel miRNAs were performed with TargetScan, which allows accessible off-line use. Human was chosen as the target organism and limit to target sites widely conserved in mammals was applied.

#### cDNA synthesis for RT-qPCR

Individual samples from all animals mentioned in the experimental infection section above were subjected to RT-qPCR study. The integrity of the total RNA ranged from 6.2-8.7. For more details see Additional file 12. 100 ng of total RNA was used for reverse transcription. A cDNA panel of duplicates for each biological sample was constructed including: 16 necrotic lung, 14 demarcation zone lung, 16 unaffected lung, 8 adult, 8 F100, 8 F50, 8 trachea and 8 nose samples (43 biological samples in duplicates making 86 cDNA samples all together). cDNA synthesis was performed as described in details in [91]. Briefly, 100 ng of RNA in a final volume of 10 μl including 1 μl of 10x poly(A) polymerase buffer, 0.1 mM of ATP, 1 μM of RT-primer (5'-CAGGTCCAGTTTTTTTTTTTTTTTVN, where V is A, C and G and N is A, C, G and T). The primer was purchased from TAG Copenhagen (Denmark), 0.1 mM of each deoxynucleotide (dATP, dCTP, dGTP and dTTP), 100 units of MuLV reverse transcriptase (New England Biolabs, USA) and 1 unit of poly(A) polymerase (New England Biolabs, USA) were incubated at 42°C for 1 hour followed by enzyme inactivation at 95°C for 5 minutes. The cDNA was diluted 8 times before used for RT-qPCR reaction.

This miR-specific qPCR method as previously described in [84], [85] depends on polyA-tailing at the 3'-end of the miRNA followed by sequence-specific PCR. However, this 3'-end is not available if the miR is located at the 5'-end of the pre-miRNA (5'-miRNA or miRNA-5p). Therefore, miR-specific qPCR only detects the mature miRNA and not the pre-miRNA for 5'-miRNAs/miRNA-5p (Busk, 2010). In contrast, when the miRNA is located at the 3'-end of the pre-miRNA (3'-miRNA or miRNA-3p) the miRNA and the pre-miRNA have identical 3'-ends. Therefore, miR-specific qPCR will detect both the miRNA and the pre-microRNA in the case of 3'-miRNAs/miRNA-3p [85].

Of the miRNAs investigated in the present study, miR15a, miR21, miR126, miR142-5p, miR144-5p, miR146a-5p, miR148a, miR152, miR155, miR192, miR-223, miR-45 and miR-d5 are 5'-miRNAs hence only the mature miRNAs of these targets were detected. For miR-143-3p, which is a 3'-miRNA both the mature miRNA and the pre-miRNA were detected.

#### Quantitative RT-PCR of microRNAs

Fourteen candidate genes (13 microRNAs and 1 snoRNA) and two reference genes (miR-152 and MiR-191) were assayed by RT-qPCR. Primer sequences for each assayed ncRNA gene (including reference genes) are listed in the Additional file 13. Semi Quantitative PCR of 86 samples was performed on a MX3000P machine (Stratagene, USA) in 10 μl total volume with 1 μl of diluted cDNA, 5 μl of 2x QuantiFast SYBR Green PCR master mix (Qiagen, Germany), 250nM of each primer ( Additional file 13). Standard curves with 5-fold dilutions (made with a pool of equal amounts of cDNA from the 88 samples) were made for all assays to calculate the RT-qPCR efficiency. Cycling conditions were 95°C for 5 min followed by 40 cycles of 95°C for 10 sec and 60°C 30 sec. A melting curve analysis (60°C to 99°C) was performed in the last cycle, to evaluate specificity of the amplification.

#### RT-qPCR data analysis

PCR efficiency was calculated from the log-linear portion of the standard curves [86]. GeneEx (MultiD) software was used to perform the analysis. Briefly, Cq values for each assayed microRNA/snoRNA were imported to GeneEx software. Data were corrected for efficiencies (ranging from 81 to 98%) for each primer assay individually, followed by the normalization of the expression of target genes to the expression of reference genes namely: miR-152 and miR-191. Technical cDNA replicates were averaged and relative quantities were calculated compared to the control group (lung samples from uninfected pigs). Prior to statistical analysis, the data was LOG2 transformed to assure normal distribution. The one factor Analysis of Variance (ANOVA) testing for significant differences between the means of the analyzed groups was performed. The confidence level was set at 95%. Tukey-Kramer's test was chosen as the post test for the all pairwise comparisons. The summary of the statistical analysis for all the sample groups is included in Additional files 8 and 14 The results are presented as a table for each gene, with sums of squares (*SS*), degrees of freedom (*df*), mean sums of squares (*MS*), F-statistics (*F*), and *p-value* (for detailed comparisons see Additional files 3 and 4). Significance threshold was set at p-value > 0.05. RT-qPCR experiment as well as the data analysis is MIQE guidelines compliant [86].

## Competing interests

The authors declare that they have no competing interests.

## Authors’ contributions

Conceived and designed the experiments: AP, SC, MF. Performed the experiments: AP. High throughput sequencing: MB, NT, AP. Conceived and designed the bioinformatics analysis: CA, JG, AP, SC, MF. Analysed the data: AP, SC, MF, CA, JG, KS. Contributed reagents/materials/analysis tools: SC, KS. Wrote the paper: AP, SC, MF, CA, JG, KS, PMHH. All authors read and approved the final manuscript.

## Supplementary Material

Additional file 1 **Profiles of read clusters for* novel* miRNAs from miR-d1 to miR-d6.** Click here for file

Additional file 2 **Profiles of read clusters for* novel* miRNAs from miR-d7 to miR-d12.** Click here for file

Additional file 3 **The known miRNAs of the miRDeep2 pipeline missed by the unique mappings from Novoalign.** 24 of these miRNAs are found twice in the genome, indicating assembly errors. Raw reads are un-normalized and according to the Bowtie alignment produced by miRDeep. Click here for file

Additional file 4 **Expression of the all the detected miRNAs.** Columns 6 and 7 contain the raw reads in the two samples. Columns 8 and 9 are the read counts normalized by the TMM method. Column 10 is log2 of the fold change based on the normalized read counts. Column 11 is the p-values for the relative expression based on the exact test from the edgeR package using a dispersion-factor of 0.1. (see Methods for details). Click here for file

Additional file 5 **Top 20 snoRNAs in the necrotic tissue.** Infernal e-values are given for the annotation. Click here for file

Additional file 6 **Top 20 snoRNAs in the unaffected tissue.** Infernal e-values are given for the annotation. Click here for file

Additional file 7 **Bar diagram showing RT-qPCR results of expression of 13 selected unique miRNAs and one snoRNA.** miR-d5 represents a novel unannotated microRNA. All eight sample groups included. Click here for file

Additional file 8 **Statistical analysis of qPCR results:One way ANOVA performed on four groups: control, visually unaffected, demarcation zone, necrotic.** Click here for file

Additional file 9 **Targets predicted for 12 select miRNAs and 91 select proteins.** Only targets predicted by two or more of the four target prediction methods (see Methods for details). Click here for file

Additional file 10 **Conservation of miRNAs found in this article.** In column 2–5 the conservation is based on full genomic pairwise alignments of pig and 17 other mammalian genomes (bosTau4, turTru1, equCab2, felCat3, canFam2, eriEur1, hg19, tarSyr1, mm9, rn4, oryCun2, loxAfr3, echTel1, dasNov2, choHof1, monDom5, ornAna1). The miRNA pig coordinates are required to transfer to the target organism, and the coordinates in the target organism is required to transfer back to the same position in pig to avoid paralogous alignments. The pig sequence is then aligned to the target sequence and a minimum align length of 50 and an identity of at least 50% is required for the miRNA to be counted in the third column. In columns 2 and 3 the pairwise identity between the pig sequence and cow or human is given, and in column 4 the number of organisms where the miRNA is found is given and finally in column 5 the mean pairwise identity is given. Columns 6–9 are analogous to column 2–5, except the identification of the miRNAs in the target organism are now identified by a BLAST of mirBase version 18 against the target genome. The pig and target sequence are then aligned in the same way as before. Click here for file

Additional file 11 **Targets predicted with TargetScan for mir-d5 and mir-d11 for which the set of conserved target sites gave few results.** The targets are predicted for 3’ UTRs from cow since the pig ones are not in the dataset for TargetScan (see Methods for details). Click here for file

Additional file 12 Table listing values describing the quantity and integrity of all RNA samples. Click here for file

Additional file 13 **Table listing RT-qPCR primer sequences for each assayed miRNA and snoRNA.** * indicates reference genes. Click here for file

Additional file 14 **Statistical analysis of qPCR results.** One way ANOVA performed on eight groups: trachea, nose, F50, F100, control, visually unaffected, demarcation zone, necrotic. Click here for file

## References

[B1] Bartel DP MicroRNAs: genomics, biogenesis, mechanism, and function Cell 2004 116 281 297 10.1016/S0092-8674(04)00045-5 14744438

[B2] Lagos-Quintana M Rauhut R Meyer J Borkhardt A Tuschl T New microRNAs from mouse and human RNA 2003 9 175 179 10.1261/rna.2146903 12554859 PMC1370382

[B3] Lim LP Glasner ME Yekta S Burge CB Bartel DP Vertebrate microRNA genes Science 2003 299 1540 10.1126/science.1080372 12624257

[B4] Ambros V The functions of animal microRNAs Nature 2004 431 350 355 10.1038/nature02871 15372042

[B5] Sayed D Abdellatif M MicroRNAs in development and disease Physiol Rev 2011 91 827 887 10.1152/physrev.00006.2010 21742789

[B6] Tomankova T Petrek M Kriegova E Involvement of microRNAs in physiological and pathological processes in the lung Respir Res 2010 11 159 10.1186/1465-9921-11-159 21092244 PMC3001429

[B7] Imler JL Hoffmann JA Toll receptors in innate immunity Trends Cell Biol 2001 11 304 311 10.1016/S0962-8924(01)02004-9 11413042

[B8] Tsiotou AG Sakorafas GH Anagnostopoulos G Bramis J Septic shock; current pathogenetic concepts from a clinical perspective Med Sci Monit 2005 11 RA76 RA85 15735579

[B9] Ding SW Voinnet O Antiviral immunity directed by small RNAs Cell 2007 130 413 426 10.1016/j.cell.2007.07.039 17693253 PMC2703654

[B10] Umbach JL Cullen BR The role of RNAi and microRNAs in animal virus replication and antiviral immunity Genes Dev 2009 23 1151 1164 10.1101/gad.1793309 19451215 PMC2763533

[B11] Lecellier CH Dunoyer P Arar K Lehmann-Che J Eyquem S Himber C Saib A Voinnet O A cellular microRNA mediates antiviral defense in human cells Science 2005 308 557 560 10.1126/science.1108784 15845854

[B12] Nathans R Chu CY Serquina AK Lu CC Cao H Rana TM Cellular microRNA and P bodies modulate host-HIV-1 interactions Mol Cell 2009 34 696 709 10.1016/j.molcel.2009.06.003 19560422 PMC2763548

[B13] Jopling CL Yi M Lancaster AM Lemon SM Sarnow P Modulation of hepatitis C virus RNA abundance by a liver-specific MicroRNA Science 2005 309 1577 1581 10.1126/science.1113329 16141076

[B14] Baltimore D Boldin MP O'Connell RM Rao DS Taganov KD MicroRNAs: new regulators of immune cell development and function Nat Immunol 2008 9 839 845 10.1038/ni.f.209 18645592

[B15] Lindsay CR Evans TJ The insulin-like growth factor system and its receptors: A potential novel anticancer target Biologics 2008 2 855 864 19707463 10.2147/btt.s3841 PMC2727903

[B16] Taganov KD Boldin MP Chang KJ Baltimore D NF-kappaB-dependent induction of microRNA miR-146, an inhibitor targeted to signaling proteins of innate immune responses Proc Natl Acad Sci USA 2006 103 12481 12486 10.1073/pnas.0605298103 16885212 PMC1567904

[B17] Tili E Michaille JJ Cimino A Costinean S Dumitru CD Adair B Fabbri M Alder H Liu CG Calin GA Croce CM Modulation of miR-155 and miR-125b levels following lipopolysaccharide/TNF-alpha stimulation and their possible roles in regulating the response to endotoxin shock J Immunol 2007 179 5082 5089 17911593 10.4049/jimmunol.179.8.5082

[B18] Ceppi M Pereira PM Dunand-Sauthier I Barras E Reith W Santos MA Pierre P MicroRNA-155 modulates the interleukin-1 signaling pathway in activated human monocyte-derived dendritic cells Proc Natl Acad Sci USA 2009 106 2735 2740 10.1073/pnas.0811073106 19193853 PMC2650335

[B19] Xiao B Liu Z Li BS Tang B Li W Guo G Shi Y Wang F Wu Y Tong WD Guo H Mao XH Zou QM Induction of microRNA-155 during Helicobacter pylori infection and its negative regulatory role in the inflammatory response J Infect Dis 2009 200 916 925 10.1086/605443 19650740

[B20] Schulte LN Eulalio A Mollenkopf HJ Reinhardt R Vogel J Analysis of the host microRNA response to Salmonella uncovers the control of major cytokines by the let-7 family EMBO J 2011 30 1977 1989 10.1038/emboj.2011.94 21468030 PMC3098495

[B21] Rycroft AN Garside LH Actinobacillus species and their role in animal disease Vet J 2000 159 18 36 10.1053/tvjl.1999.0403 10640409

[B22] Bosse JT Janson H Sheehan BJ Beddek AJ Rycroft AN Kroll JS Langford PR Actinobacillus pleuropneumoniae: pathobiology and pathogenesis of infection Microbes Infect 2002 4 225 235 10.1016/S1286-4579(01)01534-9 11880056

[B23] Mortensen S Skovgaard K Hedegaard J Bendixen C Heegaard PM Transcriptional profiling at different sites in lungs of pigs during acute bacterial respiratory infection Innate Immun 2011 17 41 53 10.1177/1753425909349760 19897530

[B24] Gorodkin J Cirera S Hedegaard J Gilchrist MJ Panitz F Jorgensen C Scheibye-Knudsen K Arvin T Lumholdt S Sawera M Green T Nielsen BJ Havgaard JH Rosenkilde C Wang J Li H Li R Liu B Hu S Dong W Li W Yu J Wang J Staefeldt HH Wernersson R Madsen LB Thomsen B Hornshoj H Bujie Z Wang X Porcine transcriptome analysis based on 97 non-normalized cDNA libraries and assembly of 1,021,891 expressed sequence tags Genome Biol 2007 8 R45 10.1186/gb-2007-8-4-r45 17407547 PMC1895994

[B25] Wernersson R Schierup MH Jorgensen FG Gorodkin J Panitz F Staerfeldt HH Christensen OF Mailund T Hornshoj H Klein A Wang J Liu B Hu S Dong W Li W Wong GK Yu J Wang J Bendixen C Fredholm M Brunak S Yang H Bolund L Pigs in sequence space: a 0.66X coverage pig genome survey based on shotgun sequencing BMC Genomics 2005 6 70 10.1186/1471-2164-6-70 15885146 PMC1142312

[B26] Kozomara A Griffiths-Jones S miRBase: integrating microRNA annotation and deep-sequencing data Nucleic Acids Res 2011 39 D152 D157 10.1093/nar/gkq1027 21037258 PMC3013655

[B27] Griffiths-Jones S Saini HK van Dongen S Enright AJ miRBase: tools for microRNA genomics Nucleic Acids Res 2008 36 D154 D158 10.1093/nar/gkn221 17991681 PMC2238936

[B28] Griffiths-Jones S Grocock RJ van Dongen S Bateman A Enright AJ miRBase: microRNA sequences, targets and gene nomenclature Nucleic Acids Res 2006 34 D140 D144 10.1093/nar/gkj112 16381832 PMC1347474

[B29] Griffiths-Jones S The microRNA Registry Nucleic Acids Res 2004 32 D109 D111 10.1093/nar/gkh023 14681370 PMC308757

[B30] Pais H Moxon S Dalmay T Moulton V Small RNA discovery and characterisation in eukaryotes using high-throughput approaches Adv Exp Med Biol 2011 722 239 254 10.1007/978-1-4614-0332-6_16 21915794

[B31] Novocraft Technologies Novoalign version 2.0.25 2011

[B32] Pig genome version 9 from ensembl At least three versions of the pig genome in circulation: ncbi version 9, ncbi version 9.2, and ensembl version 9. Ensembl version 9 is the same as ncbi version 9 except for a contig change on chr2 2011

[B33] Flicek P Amode MR Barrell D Beal K Brent S Denise CS Clapham P Coates G Fairley S Fitzgerald S Gil L Gordon L Hendrix M Hourlier T Johnson N Kahari AK Keefe D Keenan S Kinsella R Komorowska M Koscielny G Kulesha E Larsson P Longden I McLaren W Muffato M Overduin B Pignatelli M Pritchard B Riat HS Ensembl 2012 Nucleic Acids Res 2012 40 D84 D90 10.1093/nar/gkr991 22086963 PMC3245178

[B34] Altschul SF Gish W Miller W Myers EW Lipman DJ Basic local alignment search tool J Mol Biol 1990 215 403 410 2231712 10.1016/S0022-2836(05)80360-2

[B35] Nawrocki EP Kolbe DL Eddy SR Infernal 1.0: inference of RNA alignments Bioinformatics 2009 25 1335 1337 10.1093/bioinformatics/btp157 19307242 PMC2732312

[B36] Gardner PP Daub J Tate JG Nawrocki EP Kolbe DL Lindgreen S Wilkinson AC Finn RD Griffiths-Jones S Eddy SR Bateman A Rfam: updates to the RNA families database Nucleic Acids Res 2009 37 D136 D140 10.1093/nar/gkn766 18953034 PMC2686503

[B37] Gorodkin J Hofacker IL Torarinsson E Yao Z Havgaard JH Ruzzo WL De novo prediction of structured RNAs from genomic sequences Trends Biotechnol 2010 28 9 19 10.1016/j.tibtech.2009.09.006 19942311 PMC4712260

[B38] Gorodkin J Hofacker IL From structure prediction to genomic screens for novel non-coding RNAs PLoS Comput Biol 2011 7 e1002100 10.1371/journal.pcbi.1002100 21829340 PMC3150283

[B39] Friedlander MR Chen W Adamidi C Maaskola J Einspanier R Knespel S Rajewsky N Discovering microRNAs from deep sequencing data using miRDeep Nat Biotechnol 2008 26 407 415 10.1038/nbt1394 18392026

[B40] Langmead B Trapnell C Pop M Salzberg S Ultrafast and memory-efficient alignment of short DNA sequences to the human genome Genome Biol 2009 10 R25 10.1186/gb-2009-10-3-r25 19261174 PMC2690996

[B41] Gruber AR Findeiss S Waschitl S Hofacker IL Stadler PF RNAZ 2.0: IMPROVED NONCODING RNA DETECTION Pac Symp Biocomput 2010 5 69 79 Publisher: Stanford, CA: Stanford University, Department of Bioengineering 19908359

[B42] Lewis BP Burge CB Bartel DP Conserved seed pairing, often flanked by adenosines, indicates that thousands of human genes are microRNA targets Cell 2005 120 15 20 10.1016/j.cell.2004.12.035 15652477

[B43] Grimson A Farh KK Johnston WK Garrett-Engele P Lim LP Bartel DP MicroRNA targeting specificity in mammals: determinants beyond seed pairing Mol Cell 2007 27 91 105 10.1016/j.molcel.2007.06.017 17612493 PMC3800283

[B44] Friedman RC Farh KK Burge CB Bartel DP Most mammalian mRNAs are conserved targets of microRNAs Genome Res 2009 19 92 105 18955434 10.1101/gr.082701.108 PMC2612969

[B45] Lall S Grun D Krek A Chen K Wang YL Dewey CN Sood P Colombo T Bray N Macmenamin P Kao HL Gunsalus KC Pachter L Piano F Rajewsky N A genome-wide map of conserved microRNA targets in C. elegans Curr Biol 2006 16 460 471 10.1016/j.cub.2006.01.050 16458514

[B46] Krek A Grun D Poy MN Wolf R Rosenberg L Epstein EJ Macmenamin P da Piedade I Gunsalus KC Stoffel M Rajewsky N Combinatorial microRNA target predictions Nat Genet 2005 37 495 500 10.1038/ng1536 15806104

[B47] John B Enright AJ Aravin A Tuschl T Sander C Marks DS Human MicroRNA targets PLoS Biol 2004 2 e363 10.1371/journal.pbio.0020363 15502875 PMC521178

[B48] Enright AJ John B Gaul U Tuschl T Sander C Marks DS MicroRNA targets in Drosophila Genome Biol 2003 5 R1 10.1186/gb-2003-5-1-r1 14709173 PMC395733

[B49] Betel D Wilson M Gabow A Marks DS Sander C The microRNA.org resource: targets and expression Nucleic Acids Res 2008 36 D149 D153 18158296 10.1093/nar/gkm995 PMC2238905

[B50] Betel D Koppal A Agius P Sander C Leslie C Comprehensive modeling of microRNA targets predicts functional non-conserved and non-canonical sites Genome Biol 2010 11 R90 10.1186/gb-2010-11-8-r90 20799968 PMC2945792

[B51] Maragkakis M Alexiou P Papadopoulos GL Reczko M Dalamagas T Giannopoulos G Goumas G Koukis E Kourtis K Simossis VA Sethupathy P Vergoulis T Koziris N Sellis T Tsanakas P Hatzigeorgiou AG Accurate microRNA target prediction correlates with protein repression levels BMC Bioinforma 2009 10 295 10.1186/1471-2105-10-295 PMC2752464 19765283

[B52] Cirera S Nygard AB Jensen HE Skovgaard K Boye M Fredholm M Molecular characterization of the porcine surfactant, pulmonary-associated protein C gene Genomics 2006 88 659 668 10.1016/j.ygeno.2006.04.011 16769199

[B53] Kenan-Eichler M Leshkowitz D Tal L Noor E Melamed-Bessudo C Feldman M Levy AA Wheat hybridization and polyploidization results in deregulation of small RNAs Genetics 2011 188 263 272 10.1534/genetics.111.128348 21467573 PMC3122319

[B54] Rathjen T Pais H Sweetman D Moulton V Munsterberg A Dalmay T High throughput sequencing of microRNAs in chicken somites FEBS Lett 2009 583 1422 1426 10.1016/j.febslet.2009.03.048 19328789

[B55] Vaz C Ahmad HM Sharma P Gupta R Kumar L Kulshreshtha R Bhattacharya A Analysis of microRNA transcriptome by deep sequencing of small RNA libraries of peripheral blood BMC Genomics 2010 11 288 10.1186/1471-2164-11-288 20459673 PMC2885365

[B56] Nana-Sinkam SP Karsies T Riscili B Ezzie M Piper M Lung microRNA: from development to disease Expert Rev Respir Med 2009 3 373 385 10.1586/ers.09.30 20477329

[B57] Pandit KV Milosevic J Kaminski N MicroRNAs in idiopathic pulmonary fibrosis Transl Res 2011 157 191 199 10.1016/j.trsl.2011.01.012 21420029

[B58] Roy S Sen CK MiRNA in innate immune responses: novel players in wound inflammation Physiol Genomics 2011 43 557 565 10.1152/physiolgenomics.00160.2010 21139022 PMC3110889

[B59] Ma X Becker Buscaglia LE Barker JR Li Y MicroRNAs in NF-kappaB signaling J Mol Cell Biol 2011 3 159 166 10.1093/jmcb/mjr007 21502305 PMC3104013

[B60] Quinn SR O'Neill LA A trio of microRNAs that control Toll-like receptor signalling Int Immunol 2011 23 421 425 10.1093/intimm/dxr034 21652514

[B61] Karin M Greten FR NF-kappaB: linking inflammation and immunity to cancer development and progression Nat Rev Immunol 2005 5 749 759 10.1038/nri1703 16175180

[B62] Ward JR Heath PR Catto JW Whyte MK Milo M Renshaw SA Regulation of neutrophil senescence by microRNAs PLoS One 2011 6 e15810 10.1371/journal.pone.0015810 21283524 PMC3023715

[B63] Case SR Martin RJ Jiang D Minor MN Chu HW MicroRNA-21 inhibits toll-like receptor 2 agonist-induced lung inflammation in mice Exp Lung Res 2011 37 500 508 10.3109/01902148.2011.596895 21892915

[B64] Matsushima K Isomoto H Inoue N Nakayama T Hayashi T Nakayama M Nakao K Hirayama T Kohno S MicroRNA signatures in Helicobacter pylori-infected gastric mucosa Int J Cancer 2011 128 361 370 10.1002/ijc.25348 20333682

[B65] Sheedy FJ Palsson-McDermott E Hennessy EJ Martin C O'Leary JJ Ruan Q Johnson DS Chen Y O'Neill LA Negative regulation of TLR4 via targeting of the proinflammatory tumor suppressor PDCD4 by the microRNA miR-21 Nat Immunol 2010 11 141 147 10.1038/ni.1828 19946272

[B66] Anselmo A Flori L Jaffrezic F Rutigliano T Cecere M Cortes-Perez N Lefevre F Rogel-Gaillard C Giuffra E Co-expression of host and viral microRNAs in porcine dendritic cells infected by the pseudorabies virus PLoS One 2011 6 e17374 10.1371/journal.pone.0017374 21408164 PMC3050891

[B67] Rodriguez A Vigorito E Clare S Warren MV Couttet P Soond DR Van DS Grocock RJ Das PP Miska EA Vetrie D Okkenhaug K Enright AJ Dougan G Turner M Bradley A Requirement of bic/microRNA-155 for normal immune function Science 2007 316 608 611 10.1126/science.1139253 17463290 PMC2610435

[B68] O'Connell RM Taganov KD Boldin MP Cheng G Baltimore D MicroRNA-155 is induced during the macrophage inflammatory response Proc Natl Acad Sci USA 2007 104 1604 1609 10.1073/pnas.0610731104 17242365 PMC1780072

[B69] Kiorpes AL MacWilliams PS Schenkman DI Backstrom LR Blood gas and hematological changes in experimental peracute porcine pleuropneumonia Can J Vet Res 1990 54 164 169 2106382 PMC1255622

[B70] Tan Z Randall G Fan J Camoretti-Mercado B Brockman-Schneider R Pan L Solway J Gern JE Lemanske RF Nicolae D Ober C Allele-specific targeting of microRNAs to HLA-G and risk of asthma Am J Hum Genet 2007 81 829 834 10.1086/521200 17847008 PMC2227932

[B71] Collison A Herbert C Siegle JS Mattes J Foster PS Kumar RK Altered expression of microRNA in the airway wall in chronic asthma: miR-126 as a potential therapeutic target BMC Pulm Med 2011 11 29 10.1186/1471-2466-11-29 21605405 PMC3116478

[B72] Skovgaard K Mortensen S Boye M Hedegaard J Heegaard PM Hepatic gene expression changes in pigs experimentally infected with the lung pathogen Actinobacillus pleuropneumoniae as analysed with an innate immunity focused microarray Innate Immun 2010 16 343 353 10.1177/1753425909342730 19710094

[B73] Lestrade L Weber MJ snoRNA-LBME-db, a comprehensive database of human H/ACA and C/D box snoRNAs Nucleic Acids Res 2006 34 D158 D162 10.1093/nar/gkj002 16381836 PMC1347365

[B74] Juhling F Morl M Hartmann RK Sprinzl M Stadler PF Putz J tRNAdb 2009: compilation of tRNA sequences and tRNA genes Nucleic Acids Res 2009 37 D159 D162 10.1093/nar/gkn772 18957446 PMC2686557

[B75] Griffiths-Jones S Saini HK Van DS Enright AJ miRBase: tools for microRNA genomics Nucleic Acids Res 2008 36 D154 D158 10.1093/nar/gkn221 17991681 PMC2238936

[B76] Lagesen K Hallin P Rodland EA Staerfeldt HH Rognes T Ussery DW RNAmmer: consistent and rapid annotation of ribosomal RNA genes Nucleic Acids Res 2007 35 3100 3108 10.1093/nar/gkm160 17452365 PMC1888812

[B77] Lowe TM Eddy SR tRNAscan-SE: a program for improved detection of transfer RNA genes in genomic sequence Nucleic Acids Res 1997 25 955 964 9023104 10.1093/nar/25.5.955 PMC146525

[B78] Harris RS Improved pairwise alignment of genomic DNA. Ph.D. Thesis 2007 The Pennsylvania State University

[B79] Blanchette M Kent WJ Riemer C Elnitski L Smit AF Roskin KM Baertsch R Rosenbloom K Clawson H Green ED Haussler D Miller W Aligning multiple genomic sequences with the threaded blockset aligner Genome Res 2004 14 708 715 10.1101/gr.1933104 15060014 PMC383317

[B80] Gruber AR Findeiss S Washietl S Hofacker IL Stadler PF RNAZ 2.0: IMPROVED NONCODING RNA DETECTION Pac Symp Biocomput 2010 15 69 79 19908359

[B81] Scott MS Avolio F Ono M Lamond AI Barton GJ Human miRNA precursors with box H/ACA snoRNA features PLoS Comput Biol 2009 5 e1000507 10.1371/journal.pcbi.1000507 19763159 PMC2730528

[B82] Morin RD O'Connor MD Griffith M Kuchenbauer F Delaney A Prabhu AL Zhao Y McDonald H Zeng T Hirst M Eaves CJ Marra MA Application of massively parallel sequencing to microRNA profiling and discovery in human embryonic stem cells Genome Res 2008 18 610 621 10.1101/gr.7179508 18285502 PMC2279248

[B83] Haw RA Croft D Yung CK Ndegwa N D'Eustachio P Hermjakob H Stein LD The Reactome BioMart Database (Oxford) 2011 2011 bar031 22012987 10.1093/database/bar031 PMC3197281

[B84] Balcells I Cirera S Busk PK Specific and sensitive quantitative RT-PCR of miRNAs with DNA primers BMC Biotechnol 2011 11 70 10.1186/1472-6750-11-70 21702990 PMC3135530

[B85] Busk PK Method for Quantification of Small RNA Species 2010 WO/2010/085966

[B86] Bustin SA Benes V Garson JA Hellemans J Huggett J Kubista M Mueller R Nolan T Pfaffl MW Shipley GL Vandesompele J Wittwer CT The MIQE guidelines: minimum information for publication of quantitative real-time PCR experiments Clin Chem 2009 55 611 622 10.1373/clinchem.2008.112797 19246619

