# Supplementary figures and images for: Profiling microRNAs in lung tissue from pigs infected with Actinobacillus pleuropneumoniae
Source: BMC Genomics. 2012 Sep 6;13:459. doi: 10.1186/1471-2164-13-459 (PMC3465251; doi:10.1186/1471-2164-13-459)

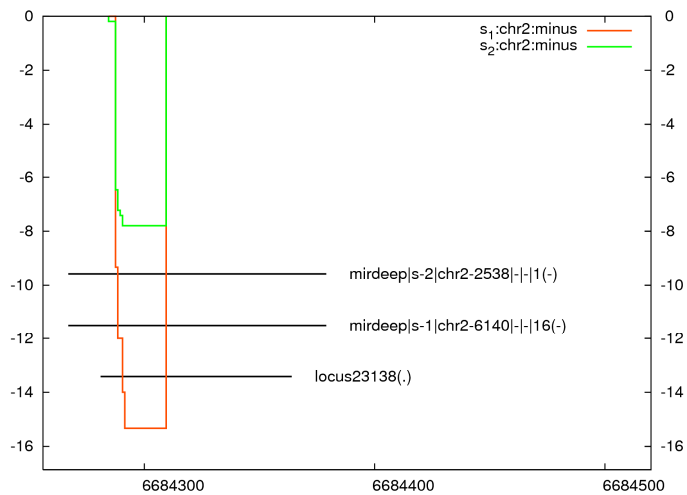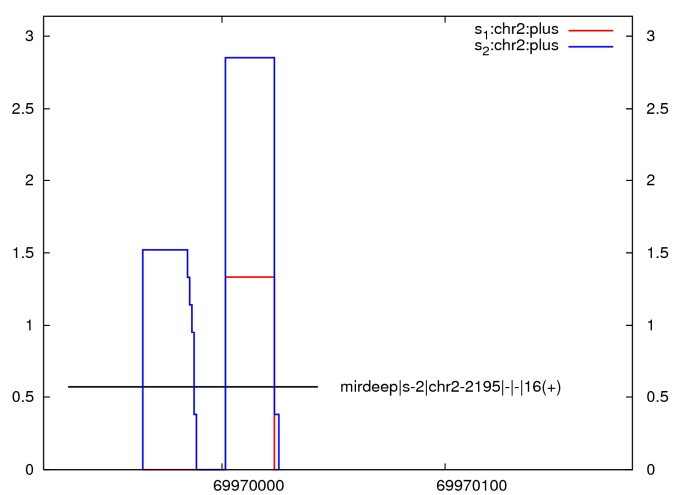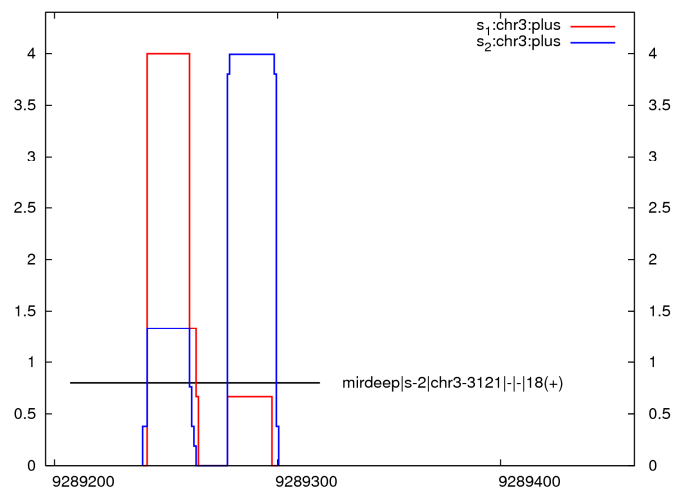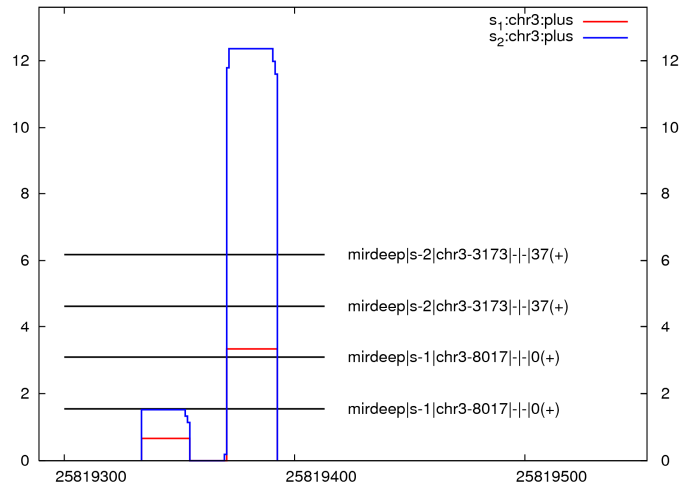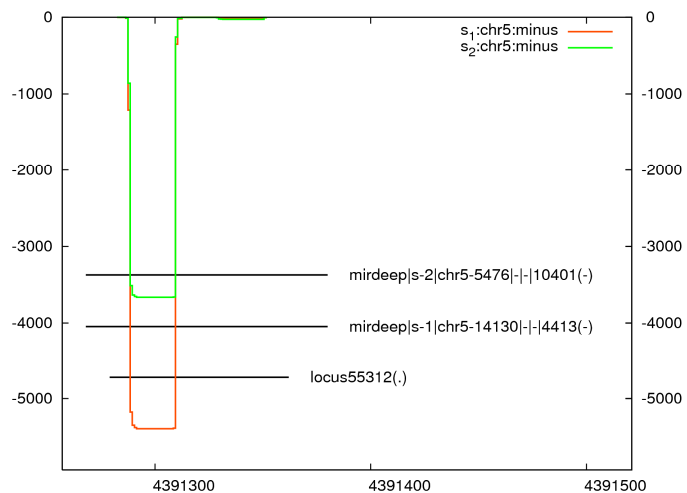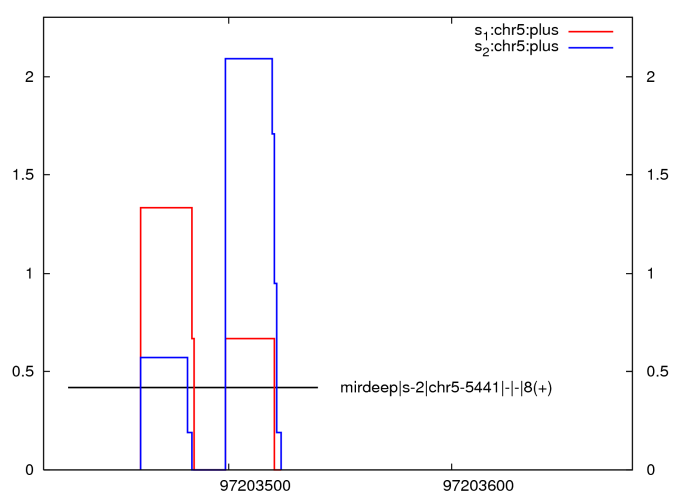

Supplement: Additional file 1 — Profiles of read clusters for novel miRNAs from miR-d1 to miR-d6. [file 1471-2164-13-459-S1.pdf]

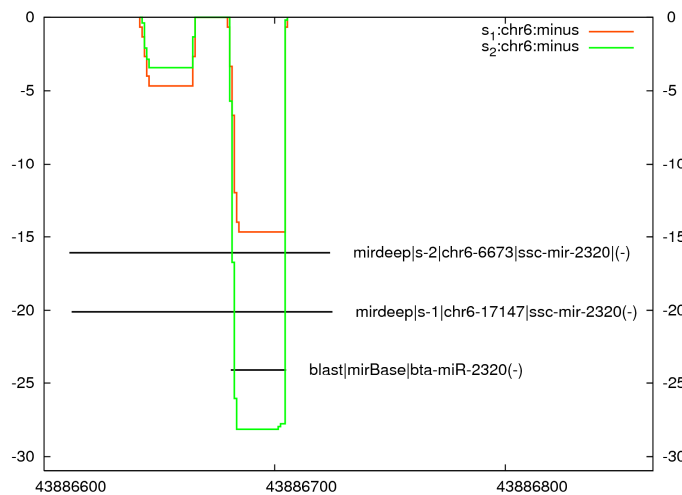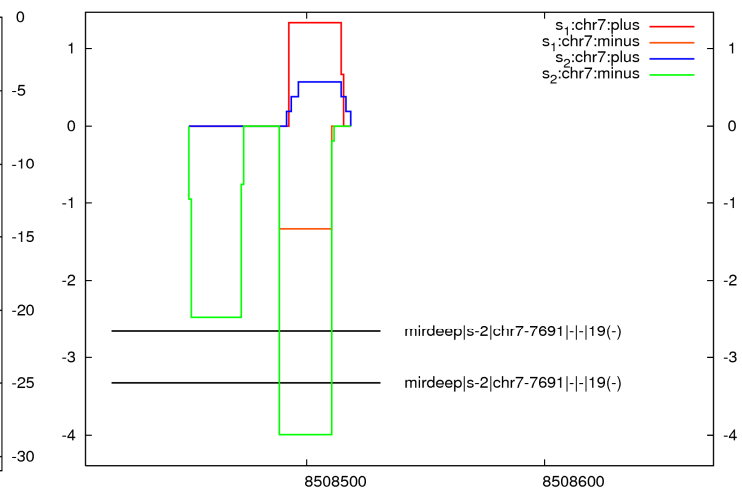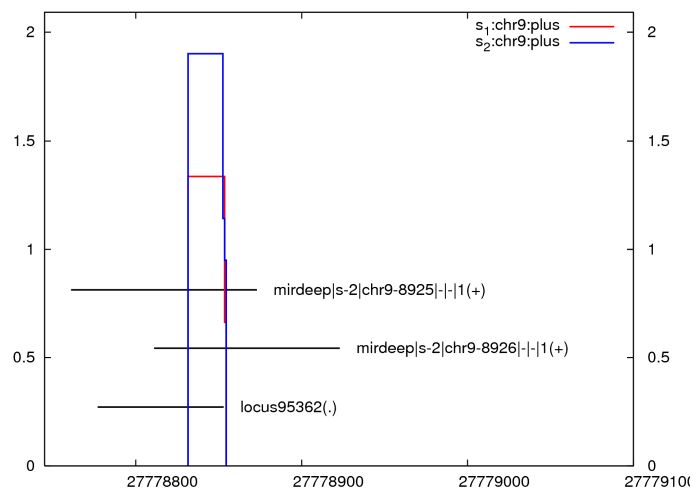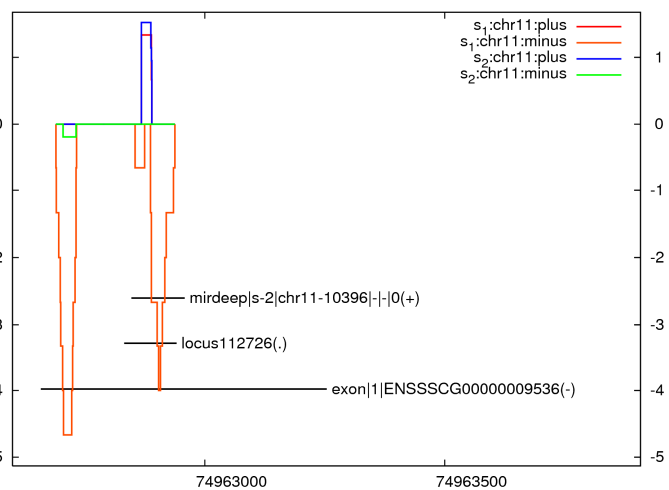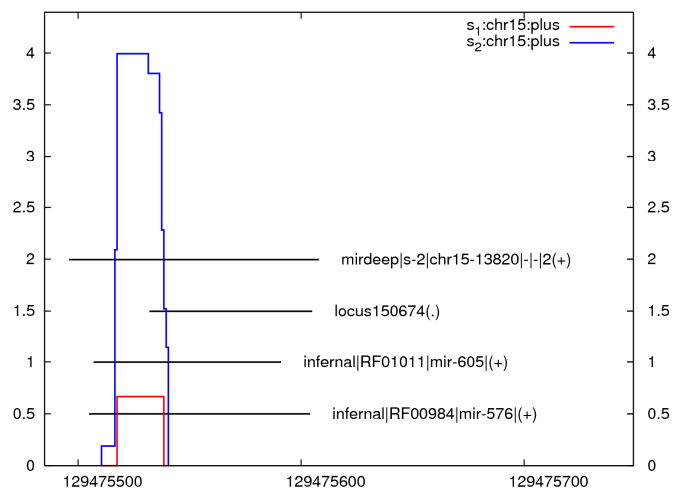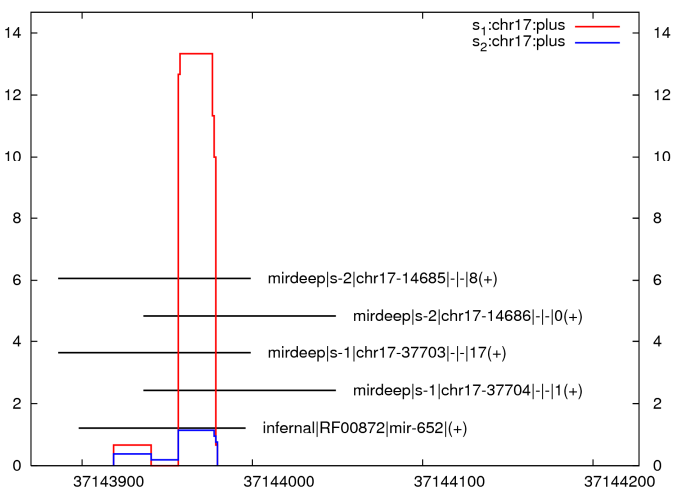

Supplement: Additional file 2 — Profiles of read clusters for novel miRNAs from miR-d7 to miR-d12. [file 1471-2164-13-459-S2.pdf]

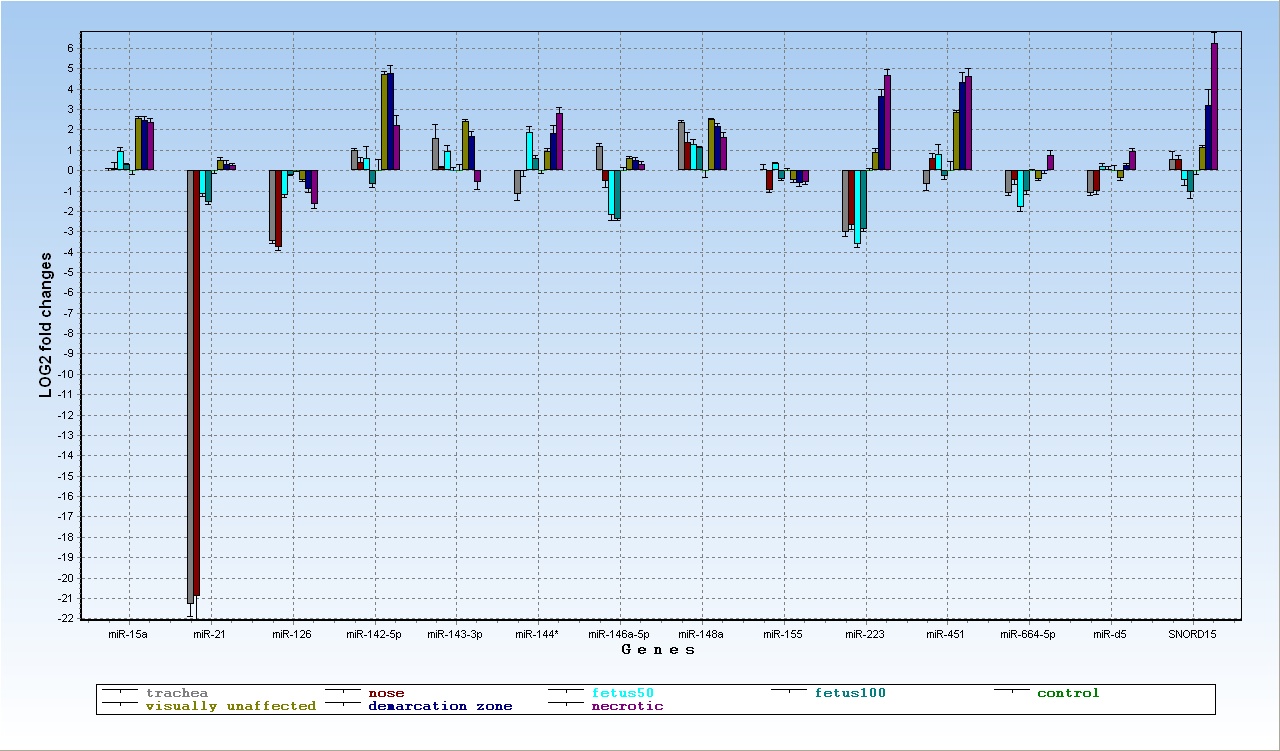

Supplement: Additional file 7 — Bar diagram showing RT-qPCR results of expression of 13 selected unique miRNAs and one snoRNA. miR-d5 represents a novel unannotated microRNA. All eight sample groups included. [file 1471-2164-13-459-S7.jpeg]
