# Supplementary material for: Profiling microRNAs in lung tissue from pigs infected with Actinobacillus pleuropneumoniae
Source: BMC Genomics. 2012 Sep 6;13:459. doi: 10.1186/1471-2164-13-459 (PMC3465251; doi:10.1186/1471-2164-13-459)
Supplement: Additional file 3 — The known miRNAs of the miRDeep2 pipeline missed by the unique mappings from Novoalign. 24 of these miRNAs are found twice in the genome, indicating assembly errors. Raw reads are un-normalized and according to the Bowtie alignment produced by miRDeep. [file 1471-2164-13-459-S3.doc]

| **Annotation** | **Coordinates** | **Normalized read counts** | |
| --- | --- | --- | --- |
|  |  | **Necrotic** | **Unaffected** |
| mir-219-2 | chr1:283451006-283451118:-,chr1:283497670-283497782:- | - | 15 |
| mir-199a-2 | chr2:52684945-52685056:- | 3380 | 24722 |
| mir-340 | chr2:67820603-67820713:+,chr2:67919920-67920028:+ | 539 | 731 |
| mir-9-2 | chr2:85597075-85597188:- | 61 | 1234 |
| mir-216 | chr3:79385086-79385198:-,chr3:79165849-79165961:+ | - | 8 |
| mir-760 | chr4:127718039-127718149:- | - | 8 |
| mir-124a-1 | chr4:72452803-72452913:- | - | 13 |
| mir-9-1 | chr4:97746771-97746884:-,chr4:97531320-97531431:+ | 61 | 1234 |
| mir-135-2 | chr5:81615619-81615732:- |  | 57 |
| mir-1249 | chr5:875639-875751:-,chr5:927581-927691:+ |  | 57 |
| mir-150 | chr6:38211379-38211490:+,chr6:38390174-38390286:- | 190 | 837 |
| mir-935 | chr6:40351560-40351671:+,chr6:40293215-40293326:+ | - | 7 |
| mir-4331 | chr6:43795775-43795886:+ | - | 299 |
| mir-345 | chr7:130435900-130436011:+,chr7:130622826-130622936:- | 31 | 190 |
| mir-493 | chr7:133858651-133858763:- | - | 14 |
| mir-133b | chr7:53115211-53115322:+ | - | 33 |
| mir-9-3 | chr7:60349120-60349231:+ | 62 | 1238 |
| mir-218 | chr8:11840683-11840793:+,chr8:12027369-12027481:- | - | 1263 |
| mir-34b | chr9:38210578-38210690:+,chr9:38327556-38327668:+ | 330 | 3720 |
| mir-34c | chr9:38211149-38211260:+,chr9:38328127-38328238:+ | 9537 | 112018 |
| mir-125b-1 | chr9:47573146-47573258:-,chr9:47654226-47654338:- | 520 | 9473 |
| mir-466i-5p | chr12:26808402-26808510:- | 350 | - |
| mir-124a-2 | chr14:13279331-13279441:- | - | 13 |
| mir-4334 | chr15:114394187-114394295:+ | - | 167 |
| mir-449b | chr16:31984166-31984276:+,chr16:31831940-31832053:- | 35 | 305 |
| mir-449 | chr16:31984287-31984397:+,chr16:31831820-31831932:- | 234 | 2192 |
| mir-218-2 | chr16:52396949-52397059:+ | 77 | 1259 |
| mir-486-1 | chr17:10710496-10710604:+,chr17:10558696-10558806:+ | 1410 | 430 |
| mir-486-2 | chr17:10710546-10710656:+,chr17:10558746-10558856:+ | 1412 | 423 |
| mir-103-2 | chr17:33574967-33575080:-,chr17:33406479-33406592:- | 3330 | 35198 |
| mir-490 | chr18:10994288-10994400:-,chr18:10924538-10924649:+ | 11 | 109 |
| mir-29b | chr18:16054328-16054438:+ | 88 | 682 |
| mir-129-1 | chr18:18269477-18269588:+ | - | 164 |
| mir-196b | chr18:44179784-44179895:+,chr18:44205838-44205949:+ | 40 | 63 |
| mir-363 | chrX:108178307-108178419:-,chrX:108212229-108212341:- | 161 | 1205 |
| mir-19b-2 | chrX:108178610-108178723:-,chrX:108212532-108212645:- | 460 | 1072 |
| mir-92a-2 | chrX:108212395-108212506:-,chrX:108178473-108178584:- | 2218 | 6263 |
| mir-20b | chrX:108212647-108212760:-,chrX:108178725-108178838:- | 53 | 323 |
| mir-676 | chrX:55113026-55113137:-,chrX:55165130-55165241:- | 19 | 122 |

Additional data file 3

The known miRNAs of the miRDeep2 pipeline missed by the unique mappings from Novoalign. 24 of these miRNAs are found twice in close the genome, indicating assembly errors. Read counts are un-normalized and according to the Bowtie alignment produced by miRDeep.
