# Supplementary material for: Profiling microRNAs in lung tissue from pigs infected with Actinobacillus pleuropneumoniae
Source: BMC Genomics. 2012 Sep 6;13:459. doi: 10.1186/1471-2164-13-459 (PMC3465251; doi:10.1186/1471-2164-13-459)
Supplement: Additional file 5 — Top 20 snoRNAs in the necrotic tissue. Infernal e-values are given for the annotation. [file 1471-2164-13-459-S5.doc]

Additional file 5

| **Annotation** | **Annotation coordinates** | **Normalized read counts** | |
| --- | --- | --- | --- |
|  |  | **Necrotic** | **Unaffected** |
| snR39B|evalue=5.61e-11 | chr13:94182263-94182332:+ | 39370 | 16587 |
| SNORD43|evalue=8.02e-07 | chr5:5237452-5237516:+ | 19461 | 6521 |
| SNORD15|evalue=1.11e-13 | chr9:9212186-9212336:- | 3695 | 60 |
| SNORND104|evalue=1.19e-11 | chr12:12437385-12437454:- | 3464 | 1288 |
| SNORD78|evalue=2.66e-09 | chr9:108992197-108992262:+ | 2838 | 1921 |
| SNORD57|evalue=9.74e-11 | chr17:34572055-34572127:- | 2618 | 1478 |
| snoU2-25|evalue=1.34e-35 | chr4:115465290-115465700:- | 2483 | 26 |
| SNORD21|evalue=1.00e-12 | chr4:128582939-128583035:- | 2018 | 157 |
| SNORA38|evalue=4.33e-24 | chr7:27701538-27701669:+ | 1467 | 3 |
| SNORD14|evalue=1.88e-08 | chr9:48526707-48526794:- | 1293 | 97 |
| SNORD38|evalue=5.82e-11 | chr6:119373197-119373266:- | 1281 | 2086 |
| SNORD20|evalue=3.14e-14 | chr15:124485678-124485758:+ | 1224 | 441 |
| SNORD74|evalue=1.54e-11 | chr9:108990369-108990447:+ | 1205 | 2448 |
| SNORD62|evalue=2.03e-13 | chr1:286820620-286820706:+ | 1194 | 76 |
| SNORD62|evalue=1.14e-12 | chr1:286816615-286816701:+ | 1179 | 102 |
| SNORD49|evalue=4.60e-11 | chr12:56174899-56174970:- | 1155 | 376 |
| SNORD89|evalue=7.45e-14 | chr3:48737839-48737949:- | 1081 | 14 |
| SNORD75|evalue=2.64e-07 | chr9:108990956-108991018:+ | 1037 | 1940 |
| SNORD100|evalue=4.32e-12 | chr1:32410910-32410987:- | 923 | 219 |
| SNORD50|evalue=2.18e-08 | chr1:57077367-57077441:- | 892 | 102 |

Top 20 snoRNAs in the necrotic tissue. Read counts in the respective samples are normalized by the number of uniquely mapped reads in read clusters with at least 5 reads minus the number of reads annotated as rRNA or protein (all per 1,000,000 reads or ~1.5 and ~5.2 respectively).
