# Supplementary material for: Profiling microRNAs in lung tissue from pigs infected with Actinobacillus pleuropneumoniae
Source: BMC Genomics. 2012 Sep 6;13:459. doi: 10.1186/1471-2164-13-459 (PMC3465251; doi:10.1186/1471-2164-13-459)
Supplement: Additional file 6 — Top 20 snoRNAs in the unaffected tissue. Infernal e-values are given for the annotation. [file 1471-2164-13-459-S6.doc]

**Additional file 6**

| **Annotation** | **Annotation coordinates** | **Normalized read counts** | |
| --- | --- | --- | --- |
|  |  | **Necrotic** | **Unaffected** |
| SNORD29|evalue=1.65e-05 | chr9:100843000-100843067:- | 197 | 19104 |
| snR39B|evalue=5.61e-11 | chr13:94182263-94182332:+ | 39370 | 16587 |
| SNORD42|evalue=9.11e-08 | chr12:42636687-42636754:- | 519 | 6769 |
| SNORD43|evalue=8.02e-07 | chr5:5237452-5237516:+ | 19461 | 6521 |
| SNORD74|evalue=1.54e-11 | chr9:108990369-108990447:+ | 1205 | 2448 |
| SNORD77|evalue=4.48e-06 | chr9:108991538-108991604:+ | 64 | 2113 |
| SNORD38|evalue=5.82e-11 | chr6:119373197-119373266:- | 1281 | 2086 |
| SNORD75|evalue=2.64e-07 | chr9:108990956-108991018:+ | 1037 | 1940 |
| SNORD78|evalue=2.66e-09 | chr9:108992197-108992262:+ | 2838 | 1921 |
| SNORD99|evalue=3.31e-05 | chr3:102062802-102062872:- | 25 | 1768 |
| SNORD57|evalue=9.74e-11 | chr17:34572055-34572127:- | 2618 | 1478 |
| SNORD81|evalue=3.79e-08 | chr9:108993771-108993850:+ | 480 | 1453 |
| SNORND104|evalue=1.19e-11 | chr12:12437385-12437454:- | 3464 | 1288 |
| SNORD45|evalue=8.03e-13 | chr6:96120921-96120992:- | 879 | 1287 |
| snoZ40|evalue=1.77e-14 | chr9:26391549-26391622:+ | 383 | 1076 |
| SNORD63|evalue=1.17e-09 | chr2:127604868-127604939:- | 825 | 977 |
| snoZ17|evalue=1.41e-06 | chr12:42633845-42633922:- | 443 | 744 |
| SNORD50|evalue=1.69e-09 | chr1:57077088-57077158:- | 806 | 566 |
| SNORD87|evalue=3.56e-12 | chr4:70340820-70340909:+ | 259 | 500 |
| SNORD18|evalue=2.11e-06 | chr1:171646170-171646242:- | 581 | 477 |

Top 20 snoRNAs in the unaffected tissue. Read counts in the respective samples are normalized by the number of uniquely mapped reads in read clusters with at least 5 reads minus the number of reads annotated as rRNA or protein (all per 1,000,000 reads or ~1.5 and ~5.2 respectively).
