# Supplementary material for: Profiling microRNAs in lung tissue from pigs infected with Actinobacillus pleuropneumoniae
Source: BMC Genomics. 2012 Sep 6;13:459. doi: 10.1186/1471-2164-13-459 (PMC3465251; doi:10.1186/1471-2164-13-459)
Supplement: Additional file 9 — Targets predicted for 12 select miRNAs and 91 select proteins. Only targets predicted by two or more of the four target prediction methods (see Methods for details). [file 1471-2164-13-459-S9.doc]

**Additional file 9**

Targets predicted for 12 select miRNAs and 91 select proteins. Only targets predicted by two or more of the four target prediction methods (see Methods for details). Targets predicted by one or more methods are found in additional file 14.

| **Gene target** | **microRNA** | **Protein function** |
| --- | --- | --- |
| CCBP2 | hsa-miR-146a | Critical for recruitment of the immune cells to the inflammation site |
| CD163 | hsa-miR-15a | Macrophage activation in inflammatory conditions. Cleaning the tissue from hemoglobin. Involved in IL6 cascade. |
| CYP26B1 | hsa-miR-155, hsa-miR-15a | Cytochrome P450, family 26, subfamily B, |
| FKBP1A | hsa-miR-142-5p, hsa-miR-15a | Role in immuno-regulation and basic cellular processes involving protein folding and trafficking |
| IL10RA | hsa-miR-15a | Receptor unit for IL10 involved in inhibition of the synthesis of pro inflammatory cytokines. |
| IL12A | hsa-miR-21 | T cell independent induction of IFNG. Lymphocytes respond to this cytokine. |
| IL13 | hsa-miR-155 | B cells maturation. Inhibition of pro inflammatory cytokines |
| IL18R1 | hsa-miR-664 | Receptor for interleukin 18 (IL-18). Binding to the agonist leads to the activation of NF-kappa-B |
| IL1A | hsa-miR-664 | Produced by activated macrophages, IL-1 stimulates thymocyte proliferation by inducing IL-2 release, B-cell maturation and proliferation, and fibroblast growth factor activity |
| IL1B | hsa-miR-21 | Produced by activated macrophages, IL-1 stimulates thymocyte proliferation by inducing IL-2 release, B-cell maturation and proliferation. |
| IL6ST | hsa-miR-223 | Receptor unit for cytokine IL6. |
| INPP5D | hsa-miR-155 | Acts as a negative regulator of B-cell antigen receptor signaling. |
| IRAK1 | hsa-miR-146a | Role in the LPS pathway. Transcription up regulation and mRNA stability. |
| MCL1 | hsa-miR-142-5p | Anti apoptotic. Regulation of apoptosis in dying cells |
| PAK2 | hsa-miR-155 | Stimulates cell survival and growth |
| PDCD4 | hsa-miR-15a  hsa-miR-21 | Present in NK and T cells. If present, inhibits helicase responsible for RNA binding to ribosomes. Present inflammatory pathways. |
| SMAD2 | hsa-miR-148a, hsa-miR-155 | Regulation of cell growth and proliferation |
| SMAD7 | hsa-miR-15a, hsa-miR-21 | Inhibits TGFbeta signaling involved in the immune response |
| SMG1 | hsa-miR-664 | mRNA surveillance and genotoxic stress response |
| SOCS5 | hsa-miR-155  hsa-miR-15a | Part of a classical negative feedback system that regulates cytokine signal transduction. |
| TAB2 | hsa-miR-155  hsa-miR-223 | Present in the IL1 and LPS inflammatory pathways |
| TAB3 | hsa-miR-15a, hsa-miR-21 | Activation of NF-kappa-B and AP1 transcription factor. |
| TERF1 | hsa-miR-155 | A telomerase specific protein which is a component of the telomere nucleoprotein complex. |
| TNFAIP1 | hsa-miR-15a, hsa-miR-664 | Possibly anti apoptotic |
| TOM1 | hsa-miR-126 | May be involved in intracellular trafficking. Probable association with membranes |
| TRAF6 | hsa-miR-146a | NF-kB activation in response to inflammatory cytokines |
