# Supplementary material for: Profiling microRNAs in lung tissue from pigs infected with Actinobacillus pleuropneumoniae
Source: BMC Genomics. 2012 Sep 6;13:459. doi: 10.1186/1471-2164-13-459 (PMC3465251; doi:10.1186/1471-2164-13-459)
Supplement: Additional file 11 — Targets predicted with TargetScan for mir-d5 and mir-d11 for which the set of conserved target sites gave few results. The targets are predicted for 3’ UTRs from cow since the pig ones are not in the dataset for TargetScan (see Methods for details). [file 1471-2164-13-459-S11.docx]

Additional data file 11

Best cow protein targets predicted by targetscan for miR-d5 and miR-d11. Conservation of the target sites not considered.

| miRNA | Protein name (human) | Targetscan score |
| --- | --- | --- |
| miR-d5 | CSDA | -0.4292 |
| miR-d5 | FAM190B | -0.4091 |
| miR-d5 | NPTN | -0.3911 |
| miR-d5 | DPM1 | -0.3775 |
| miR-d5 | BNIP3L | -0.37115 |
| miR-d5 | RGPD6 | -0.3643 |
| miR-d5 | DIRAS1 | -0.3611 |
| miR-d5 | INHBA | -0.3292 |
| miR-d5 | USP45 | -0.3085 |
| miR-d5 | MDGA2 | -0.294 |
| miR-d5 | GDAP1 | -0.2842 |
| miR-d5 | PDSS2 | -0.284 |
| miR-d5 | CLTC | -0.275 |
| miR-d5 | TDP1 | -0.27 |
| miR-d5 | HPCA | -0.2623 |
| miR-d5 | MN1 | -0.2622 |
| miR-d5 | TBX19 | -0.259 |
| miR-d5 | H2AFJ | -0.2572 |
| miR-d5 | EPB41L3 | -0.2495 |
| miR-d5 | C5orf30 | -0.2435 |
| miR-d5 | DDX6 | -0.2432 |
| miR-d5 | ZNF580 | -0.2425 |
| miR-d5 | CLDN16 | -0.238 |
| miR-d5 | AP3B1 | -0.2375 |
| miR-d5 | SLC10A7 | -0.232 |
| miR-d11 | ZFR | -0.54325 |
| miR-d11 | ZPBP2 | -0.5062 |
| miR-d11 | FOXO3 | -0.50415 |
| miR-d11 | ZBTB11 | -0.5033 |
| miR-d11 | MARK1 | -0.49925 |
| miR-d11 | DNAJC19 | -0.4992 |
| miR-d11 | EGF | -0.4931 |
| miR-d11 | SPIN1 | -0.4872 |
| miR-d11 | CCDC67 | -0.48325 |
| miR-d11 | SHOC2 | -0.4823 |
| miR-d11 | C8orf42 | -0.48215 |
| miR-d11 | SLCO4A1 | -0.4781 |
| miR-d11 | RRM2B | -0.4692 |
| miR-d11 | ZFP30 | -0.45725 |
| miR-d11 | PELI2 | -0.45615 |
| miR-d11 | MAP2 | -0.4552 |
| miR-d11 | C1orf109 | -0.4533 |
| miR-d11 | SIM2 | -0.44715 |
| miR-d11 | NEDD4 | -0.4305 |
| miR-d11 | HDGF | -0.42725 |
| miR-d11 | CNTN4 | -0.42025 |
| miR-d11 | KIF24 | -0.4193 |
| miR-d11 | PMEPA1 | -0.4102 |
| miR-d11 | USP6NL | -0.3942 |
| miR-d11 | AKAP2 | -0.3881 |
| miR-d11 | SOAT1 | -0.3733 |
| miR-d11 | ERBB3 | -0.36815 |
| miR-d11 | ETF1 | -0.36715 |
| miR-d11 | C7orf60 | -0.3583 |
| miR-d11 | DGAT2 | -0.358 |
| miR-d11 | ZCCHC3 | -0.357 |
| miR-d11 | ARL8B | -0.3552 |
| miR-d11 | ST8SIA5 | -0.3482 |
| miR-d11 | SHE | -0.34225 |
| miR-d11 | LONP1 | -0.3415 |
| miR-d11 | CFL2 | -0.33925 |
| miR-d11 | KPNA1 | -0.3343 |
| miR-d11 | TLL2 | -0.333 |
