# Supplementary material for: Profiling microRNAs in lung tissue from pigs infected with Actinobacillus pleuropneumoniae
Source: BMC Genomics. 2012 Sep 6;13:459. doi: 10.1186/1471-2164-13-459 (PMC3465251; doi:10.1186/1471-2164-13-459)
Supplement: Additional file 12 — Table listing values describing the quantity and integrity of all RNA samples. [file 1471-2164-13-459-S12.doc]

**Additional data file 12**

Table listing values describing the quantity and integrity of all RNA samples.

| **Sample** | **OD [ng/μl]** | **Integrity measure (RIN*- Bioanalyzer, RQI-Experion)** | **Platform used (Bioanalyzer, Experion)** |
| --- | --- | --- | --- |
| Trachea 28 | 172,45 | 9.3 | Experion |
| Trachea 29 | 232,44 | 9.1 | Experion |
| Trachea 33 | 161,91 | 9.3 | Experion |
| Trachea 34 | 241,15 | 9.4 | Experion |
| Nose 26 | 291,4 | 8.3 | Experion |
| Nose 28 | 351,75 | 6.8 | Experion |
| Nose 32 | 124,98 | 7.2 | Experion |
| Nose 33 | 566,77 | 9.4 | Experion |
| Fetus 50 1 | 452,11 | 9.9 | Experion |
| Fetus 50 2 | 408,77 | 10.0 | Experion |
| Fetus 50 4 | 347,67 | 9.7 | Experion |
| Fetus 50 5 | 573,94 | 9.7 | Experion |
| Fetus 100 1 | 274,62 | 9.8 | Experion |
| Fetus 100 2 | 179,49 | 9.8 | Experion |
| Fetus 100 4 | 412,34 | 7.4 | Experion |
| Fetus 100 5 | 414,56 | 9.5 | Experion |
| Control SOA | 227,83 | 8.0 | Experion |
| Control SOB | 346,25 | 8.6 | Experion |
| Control SOC | 358,56 | 8.0 | Experion |
| Control 208 | 403,74 | NA | Experion |
| Necrotic 26 | 610,86 | 7.3* ; 8.2 | Bioanalyzer, Experion |
| Necrotic 29 | 539,33 | 7.1* ; 8.3 | Bioanalyzer, Experion |
| Necrotic 30 | 721,22 | 7.3* ; 8.5 | Bioanalyzer, Experion |
| Necrotic 31 | 394,99 | 7.3* ; 8.8 | Bioanalyzer, Experion |
| Necrotic 32 | 925,33 | 6.3* ; 8.5 | Bioanalyzer, Experion |
| Necrotic 33 | 331,25 | 6.7* ; 9.7 | Bioanalyzer, Experion |
| Necrotic 34 | 552,48 | 6.8* | Bioanalyzer |
| Necrotic 36 | 552,73 | 6.2* ; 8.3 | Bioanalyzer, Experion |
| Demarcation 28 | 974,03 | 9.2 | Experion |
| Demarcation 30 | 1509,46 | 9.3 | Experion |
| Demarcation 31 | 1095,53 | 9.2 | Experion |
| Demarcation 32 | 615,10 | 9.6 | Experion |
| Demarcation 33 | 1558,56 | 9.8 | Experion |
| Demarcation 34 | 668,71 | 9.9 | Experion |
| Demarcation 36 | 431,32 | 9.1 | Experion |
| Unaffected 26 | 2417,07 | 7.4* ; 9.2 | Bioanalyzer, Experion |
| Unaffected 26 | 2953,00 | 6.6* | Bioanalyzer |
| Unaffected 29 | 2287,49 | 8.0* ; 9.2 | Bioanalyzer, Experion |
| Unaffected 30 | 2246,45 | 8.0* ; 8.4 | Bioanalyzer, Experion |
| Unaffected 31 | 1802,85 | 7.4* ; 9.0 | Bioanalyzer, Experion |
| Unaffected 32 | 2127,45 | 8.7* ; 7.8 | Bioanalyzer, Experion |
| Unaffected 33 | 1537,68 | 8.7* ; 9.3 | Bioanalyzer, Experion |
| Unaffected 34 | 1677,92 | 8.4* ; 9.9 | Bioanalyzer, Experion |
| Unaffected 35 | 1351,22 | 8.3* ; 9.2 | Bioanalyzer, Experion |
| Unaffected 36 | 1506,18 | 8.5* ; 9.9 | Bioanalyzer, Experion |
