# Supplementary material for: Profiling microRNAs in lung tissue from pigs infected with Actinobacillus pleuropneumoniae
Source: BMC Genomics. 2012 Sep 6;13:459. doi: 10.1186/1471-2164-13-459 (PMC3465251; doi:10.1186/1471-2164-13-459)
Supplement: Additional file 13 — Table listing RT-qPCR primer sequences for each assayed miRNA and snoRNA. * indicates reference genes. [file 1471-2164-13-459-S13.doc]

| **microRNA** | **microRNA sequence** | **Forward primer** | **Reverse primer** | **Organism** |
| --- | --- | --- | --- | --- |
| mirR-15a | TAGCAGCACATAATGGTTTGT | cagtagcagcacataatggt | ggtccagtttttttttttttttacaa | *Sus scrofa* |
| miR-21 | TAGCTTATCAGACTGATGTTGA | tcagtagcttatcagactgatg | cgtccagtttttttttttttttcaac | *Sus scrofa* |
| miR-126 | TCGTACCGTGAGTAATAATGCG | gcagtcgtaccgtgagtaa | ccagtttttttttttttttcgcat | *Sus scrofa* |
| miR-142-5p | CATAAAGTAGAAAGCACTACT | gcagcataaagtagaaagcac | ggtccagtttttttttttttttagtag | *Sus scrofa* |
| miR-143-3p | TGAGATGAAGCACTGTAGCTC | cagtgagatgaagcactgt | tccagtttttttttttttttgagc | *Sus scrofa* |
| miR-144* | GGATATCATCATATACTGTAAGT | cgcagggatatcatcatatact | ggtccagtttttttttttttttacttac | *Homo sapiens* |
| miR-146a-5p | TGAGAACTGAATTCCATGGGTT | cagtgagaactgaattccatg | ggtccagtttttttttttttttaacc | *Homo sapiens* |
| miR-148a | TCAGTGCACTACAGAACTTTGT | agtcagtgcactacagaac | gtccagtttttttttttttttacaaag | *Sus scrofa* |
| miR-152**§** | TCAGTGCATGACAGAACTTGG | gtcagtgcatgacagaac | tccagtttttttttttttttccaag | *Sus scrofa* |
| miR-155 | TTAATGCTAATTGTGATAGGGG | cgcagttaatgctaattgtg | cagtttttttttttttttcccctatc | *Homo sapiens* |
| miR-191**§** | CAACGGAATCCCAAAAGCAGCTG | aacggaatcccaaaagca | tccagtttttttttttttttcagc | *Sus scrofa* |
| miR-223 | TGTCAGTTTGTCAAATACCCCA | cgcagtgtcagtttgtca | ccagtttttttttttttttggggta | *Homo sapiens* |
| miR-451 | AAACCGTTACCATTACTGAGTT | gcagaaaccgttaccattact | gtccagtttttttttttttttaactca | *Sus scrofa* |
| miR-664-5p | CAGGCTAGGAGAAGTGATTGGAT | gcaggctaggagaagtg | tccagtttttttttttttttatccaatc | *Sus scrofa* |
| miR-d5 | TCCGTATCCGCAGGTTCCGCA | tccgtatccgcaggt | cagtttttttttttttttgcggaa | *Sus scrofa* |
| SNORD15 | TCAATGACGAGAAGATGACGAGTCTGACTGGA | caatgacgagaagatgacga | agtttttttttttttttccagtcag | *Sus scrofa* |

**Additional data file 13**

Table listing RT-qPCR primer sequences for each assayed miRNA and snoRNA. **§** indicates reference genes
